# A statistical score for assessing the quality of multiple sequence alignments

**DOI:** 10.1186/1471-2105-7-484

**Published:** 2006-11-03

**Authors:** Virpi Ahola, Tero Aittokallio, Mauno Vihinen, Esa Uusipaikka

**Affiliations:** 1 Biotechnology and Food Research, MTT Agrifood Research Finland, Jokioinen, Finland; 2 Department of Statistics, University of Turku, Turku, Finland; 3 Department of Mathematics, University of Turku, Turku, Finland; 4 Institute of Medical Technology, University of Tampere, Tampere, Finland; 5 Research Unit, Tampere University Hospital, Tampere, Finland; 6 Systems Biology Unit, Institut Pasteur, Paris, France

## Abstract

**Background:**

Multiple sequence alignment is the foundation of many important applications in bioinformatics that aim at detecting functionally important regions, predicting protein structures, building phylogenetic trees etc. Although the automatic construction of a multiple sequence alignment for a set of remotely related sequences cause a very challenging and error-prone task, many downstream analyses still rely heavily on the accuracy of the alignments.

**Results:**

To address the need for an objective evaluation framework, we introduce a statistical score that assesses the quality of a given multiple sequence alignment. The quality assessment is based on counting the number of significantly conserved positions in the alignment using importance sampling method in conjunction with statistical profile analysis framework. We first evaluate a novel objective function used in the alignment quality score for measuring the positional conservation. The results for the Src homology 2 (SH2) domain, Ras-like proteins, peptidase M13, subtilase and *β*-lactamase families demonstrate that the score can distinguish sequence patterns with different degrees of conservation. Secondly, we evaluate the quality of the alignments produced by several widely used multiple sequence alignment programs using a novel alignment quality score and a commonly used sum of pairs method. According to these results, the Mafft strategy L-INS-i outperforms the other methods, although the difference between the Probcons, TCoffee and Muscle is mostly insignificant. The novel alignment quality score provides similar results than the sum of pairs method.

**Conclusion:**

The results indicate that the proposed statistical score is useful in assessing the quality of multiple sequence alignments.

## Background

A wealth of molecular data concerning the linear structure of proteins and nucleic acids is available in the form of DNA, RNA and protein sequences. Multiple sequence alignment has become an essential and widely used tool for understanding the structure and function of these molecules. The results of annotation of gene/protein sequences, prediction of protein structures or building of phylogenetic trees, for instance, are critically dependent on the quality of the given alignment. It has been recognized that the automatic construction of a multiple sequence alignment for a set of remotely related sequences can be a very demanding task. Therefore, there is a need for an objective approach to evaluate the alignments produced by alignment programs.

Two popular measures for scoring entire multiple alignments are the sum of pairs (SP) score and the column score (CS) [1]. These scores can, however, only be used if a reference alignment of the same sequences is available. The SP score calculates the proportion of identically aligned residue pairs in the test and the reference alignments, whereas the CS score measures the fraction of identically aligned positions. Several modifications have been made to the SP score [2,3]. The APDB (Analyze alignments with PDB) quality measure evaluates the quality of an alignment by using available tertiary structures of the sequences in the alignment [4]. The recently introduced multiple overlap score (MOS) is a promising approach, which does not need a reference alignment [5]. The MOS searches for identically aligned regions in many alignments and presumes that the alignment with the highest number of such residues also has the highest quality.

We introduce a statistical alignment quality score which first quantifies the degree of conservation at each alignment position and then counts the number of significantly conserved positions over the alignment. For measuring the degree of conservation, we use a type of *Z*-score that is based on profile analysis [6]. After deriving the maximum *Z*-score for positional conservation, the statistical significance of an observed score value is estimated using the importance sampling method [7]. The full alignment quality score is defined in terms of positional significance levels, where the multiple comparison problem is addressed with false discovery rates (FDR) [8]. The practical performance of the maxZ score is demonstrated using the SH2 domain, Ras-like proteins, peptidase M13, subtilase and *β*-lactamase families. The alignment quality score is finally applied to evaluate the alignment programs Clustal [9], TCoffee [10], Dialign2 [11], Probcons [12], Muscle [13], and Mafft [14,15].

### Related work

Several approaches have been proposed for the conservation analysis of multiple sequence alignments to quantify the degree of conservation at each aligned position using column-specific score values [16]. Valdar reviewed a wide range of such score types developed during the last two decades for protein sequence analysis [17]. He also introduced the following three criteria that a positional conservation score should fulfill: (i) the score should be a mathematical mapping from an alignment position into a bounded interval of real values which (ii) takes into account the relative symbol frequencies in the column, and (iii) their stereo-chemical properties. Additional requirements for a good conservation score include the possibility to incorporate (iv) the effect of gaps and (v) sequence weighting into (vi) a simple scoring strategy.

Existing positional scoring approaches can be roughly divided into two categories with respect to the second and third criteria. In the first category, the positional conservation is characterized based on the symbol frequencies only. Such frequency-based methods include, for instance, the information-content score that quantify the variability among the observed symbols at a particular position by means of Shannon's entropy [18,19]. A popular variation of the information-content (IC) score measures the Kullback-Leibler distance (relative entropy) between the observed symbol distribution and a background distribution of *a priori *symbol probabilities [20]. The background probability of an individual symbol may be calculated from the complete alignment, possibly supplemented with symbol-dependent pseudo-counts [21]. Alternatively, *a priori *distribution can be determined using overall relative frequencies of symbols within the sequences of the organism or protein family under investigation.

In the second category of scoring approaches, the positional conservation is characterized based on both symbol frequencies and their similarity properties. Such similarity-based scores address the fact that some symbol combinations occur more frequently than others mainly because of the chemical and physical properties. The most straightforward strategy is to group all the symbols according to their physicochemical properties before applying a particular scoring scheme. For instance, Taylor presented a classification of amino acids based on their synthesis in the Dayhoff mutation data matrix [22,23]. Subsequently, the degree of positional conservation with respect to each overlapping group of symbols can be quantified using any frequency-based scoring approach, such as the information content [24]. Different conservation scores accounting for the stereochemical sensitivity can be obtained using different symbol properties [25].

In general, the symbol properties can be considered by predefining an appropriate matrix where entries represent the similarity or dissimilarity between a symbol pair. Frequently used symbol scoring matrices for amino acids include the BLOSUM and Gonnet series of substitution matrices and PAM distance matrices [26-28]. Perhaps the most widely used scoring approach, 'sum-of-pairs', characterizes the positional conservation by calculating the sum of all pairwise similarities between the symbols in the particular column [29]. It should be noted, that this 'sum of pairs' score is different from the SP score mentioned earlier in the Background section. The SP score in [1] is used to measure alignment quality with respect to the reference alignment, whereas the score by Carillo and Lipman [29] is more generally applicable. In this work, we only use the reference alignment-based SP score. A similar but more complex mean distance (MD) score is used as an objective function in the multiple alignment software Clustal [9]. This normalized MD score also considers the fraction of gaps [30]. A number of variations can be made by using different similarity matrices on symbols or weighting schemes on sequences [31].

The present work is a continuation of our previous work on a statistical (Dunn-Sidak) framework for detecting conserved residues in the positions of a multiple sequence alignment [32]. Here, we allow for the incorporation of any symbol similarity matrix into the framework that was based on simple frequency-based scoring function. We have previously demonstrated the usefulness of this score in the automatic detection of the conserved residues in a multiple sequence alignment, and compared its results on the SH2 domain with functionally and structurally important positions of the alignment [32]. Another application of the conservation scores includes the improvement of the reliability of HMMs in the sequence similarity search by decreasing the number of false positive search results [33]. In the present study, the emphasis is on positional conservation rather than on individual residues with the aim of assessing the quality of full alignment.

## Results

### Evaluating the maxZ score for positional conservation

In this section, we study the practical performance of the maxZ score in SH2 domain, Ras-like proteins, peptidase M13, subtilase and *β*-lactamase familes. We first demonstrate the effect of five different scoring matrices and then we compare the performance of maxZ score with those of information content (IC) and Mean Distance (MD) score [20,9]. Finally, we demonstrate how the maxZ score can be used to generate a consensus sequence.

#### Multiple sequence alignments

We used the multiple sequence alignments of the SH2 domains, Ras-like proteins, peptidase M13, subtilase and *β*-lactamase families to evaluate the maxZ score. The alignments for the SH2 domain, peptidase M13, subtilase and *β*-lactamase families were obtained from the Pfam database [34]. The seed alignments of the SH2 domain, peptidase M13, subtilases and *β*-lactamases consist of 58, 24, 45 and 128 sequences, respectively. These alignments also include gaps. The sequence alignment of the Ras-like proteins was downloaded from the web page of an article by Oliveira *et al*. [35]. The alignment was build with a two-step alignment procedure [36]. First they classified sequences into groups with approximately 90% pairwise sequence identity. Sequences within each subgroup were aligned against the profile, then the groups were aligned, excluding positions with low sequence identity. The positions with gaps were also excluded from the final alignment. We used only the first sequence of each subgroup in order to avoid over-representation of profiles with many very similar sequences. This was necessary because the current maxZ score does not take the pairwise identity of the sequences into account or otherwise weight the sequences. The alignment of Ras-like proteins consists of 334 sequences.

**Figure 1:**
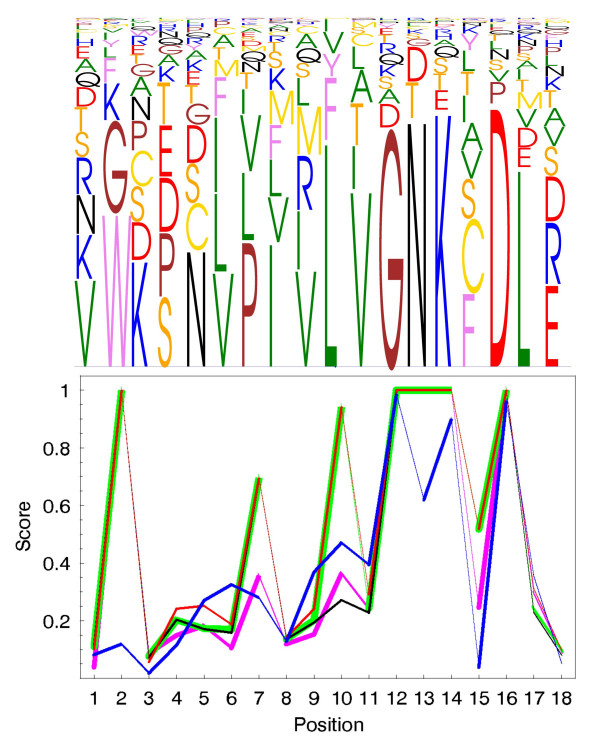
**MultiDisp visualization of part of the Ras-like proteins (upper) and the corresponding scaled -log(p)-values (lower)**. The curves show the *p*-values calculated using (red) Blosum62, (green) Gonnet250, (black) PAM250, (magenta) identity scoring matrices and (blue) classification of the amino acids for the Ras-like proteins.

**Figure 2:**
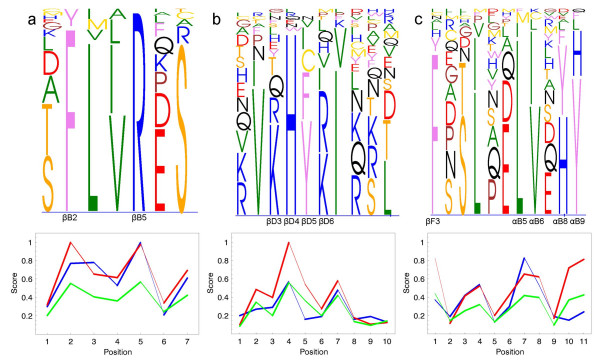
**MultiDisp visualization of the a) *βB*-stand, b) *βD*-stand and c) *αB*-helix of the SH2 domain (upper) and the corresponding conservation scores (lower)**. The curves show (red) the scaled -log(*p*)-values, (blue) Mean Distance and (green) Information content scores for the alignment. Consensus sequence for the alignment positions in c) is F P S L P E L V E H Y.

**Figure 3:**
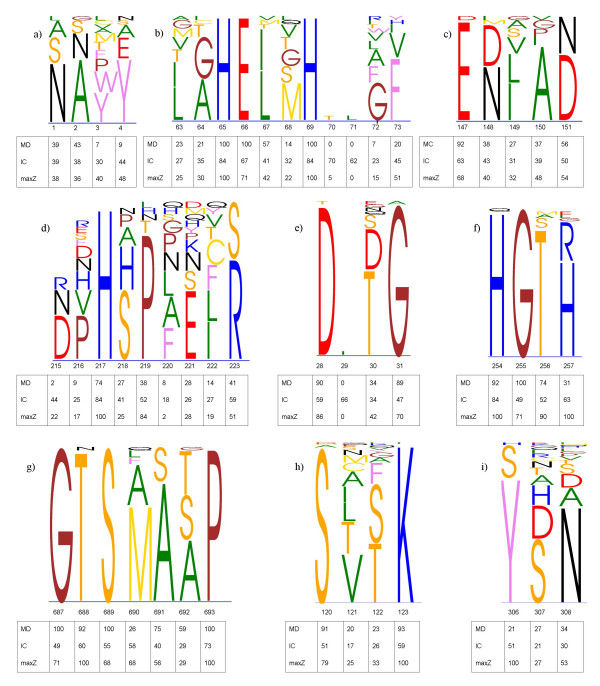
**MultiDisp visualization of the a) I, b) II, c) III and d) IV motifs of the peptidase M13, e) I, f) II and g) III motifs of the subtilase, and h) I and i) II motifs of the *β*-lactamase families and the table of the conservation scores**. MD = mean distance, IC = information content scores and maxZ = scaled -log(*p*)-values for the alignment.

Upper panels of the Figures 1, 2, 3 illustrate parts of the alignments of the Ras-like proteins, SH2 domain, peptidase M13, subtilase and *β*-lactamase families. The complete alignments of the Ras-like proteins and SH2 domain can be found as additional files (Additional files 1, 2, 3, 4, 5, 6, 7, 8, 9). The figures were generated using MultiDisp graphics program developed to visualize multiple sequence alignments [37] (Riikonen *et al*., in preparation). The lower parts of the alignments include the maxZ, MD and IC score values. The Blosum62 and grouping of amino acids were used as a scoring matrix in the maxZ score.

#### Effect of the scoring matrices

One advantage of the maxZ score is that it can consider the physicochemical relationships of amino acids. The user is able to choose an arbitrary scoring matrix or classification of the amino acids, which can be incorporated into the calculation of the maxZ score. In addition to the identity matrix, we demonstrate the use of three different scoring matrices: Blosum62, Gonnet250 and PAM250 [26-28]. Additionally, we classify amino acids into six physicochemically related groups as follows: hydrophobic {V, I, L, F, M, W, Y, C}, negatively charged {D, E}, positively charged {R, K}, conformational {G, P }, polar {N, Q, S} and {A, T}. This classification has been used, for example, by Shen and Vihinen [38]. Figure 1 shows the scaled -log(*p*)-values for the Ras-like proteins using the five different scoring schemata.

The residue positions in the alignment of Ras-like proteins were divided into five groups according to the entropy and variability [35]. The parameter values of the classification algorithm were chosen such that the groups represent the known structural and/or functional roles of the residue positions. A rough overview of the categories is the following:

• Box 11 contains positions with low entropy and variability. The positions in this group form a main functional site.

• Box 12 consists of positions with low variability and moderate entropy. These positions are located in the core of the structure next to the residues in Box 11.

• Box 22 contains positions with moderate entropy and variability. These residue positions are located in the core structure but are not adjacent to the residues in the Box 11. The positions are involved in the structure of the protein, but also in signal transmission between the modulators and the main functional site.

• Box 23 consists of the positions with high entropy and moderate variability. These positions are located at the surface or in the core of the protein and are involved in modulator interaction.

• Box 33 contains highly variable positions with high entropy. These positions are mainly located at the surface of the protein.

For a more detailed description of the categories, see the original paper [35]. Table 1 shows the median (lower and upper quartile) values of the -log(*p*)-values of the maxZ scores with different scoring matrices, along with MD and IC scores in each of the five groups. As expected, all conservation scores decreased gradually when moving from the positions with low entropy and variability to those with high entropy and variability. The performance of the MD and maxZ scores was very similar. The maxZ score with groups of amino acids distinguished slightly better than the other scores the moderately conserved positions (Boxes 12–23) from the highly conserved positions (Box 11) and unconserved ones (Box 33) (Table 1, Figure 1).

In both Ras-like protein and SH2 domain examples, all the scoring schemes tend to provide very similar results (see Additional files 1, 2, 3, 4, 5, 6, 7, 8, 9 for Blosum62 and grouping of amino acids). The results with Blosum, Gonnet and PAM matrices all rely heavily on the diagonal values of the scoring matrices. For instance, a position with highly or moderately conserved leucine obtains a relatively low maxZ score (Figure 1), whereas a position with an unconserved cysteine may be also assigned as highly conserved. This is especially critical when the Gonnet scoring matrix is used. The results with six amino acid groups differed most from the other scoring schemes since this calculates the maxZ score for the amino acid classes instead of single residues. The grouping of amino acids tends to give high scores for the positions where the majority of the residues belong to the same class. The use of the identity matrix corresponds to the special case where similarities among the symbols are ignored, and the amino acids are handled as if they where unrelated. The corresponding score is thus based solely on the relative frequencies of the residues and background probabilities. The scoring based on the identity matrix shows quite similar results with the Blosum62 and Gonnet matrices. For some positions, however, the identity matrix fails to detect the conserved positions. Similar behavior was seen with the PAM matrix (Figure 1, position 10).

**Table 1:** Median (lower and upper quartiles) of the -log(p)-values with different residue scoring schema together with the MD and IC scores.

Score	Box11	Box12	Box22	Box23	Box33
LogP Blosum62	708 (708, 708)	611 (198, 708)	208 (161, 547)	120 (99, 177)	75 (47, 123)
LogP Gonnet	708 (708, 708)	190 (164, 708)	158 (131, 189)	98 (78, 136)	64 (56, 106)
LogP Indep	708 (708, 708)	202 (158, 708)	171 (108, 202)	75 (63, 113)	57 (35, 96)
LogP PAM	708 (212, 708)	201 (166, 708)	153 (125, 201)	94 (81, 133)	66 (56, 105)
LogP 6 groups	644 (631, 683)	312 (300, 333)	279 (241, 341)	216 (77, 240)	43 (26, 91)
MD	92 (86, 97)	43 (29, 55)	34 (24, 42)	24 (19, 31)	20 (15, 25)
IC	57 (55, 59)	39 (34, 48)	31 (27, 35)	21 (19, 23)	13 (10, 19)

Box11 and Box33 represent positions with low and high entropy and variability, respectively. The three middle columns represent the moderately conserved positions. More detailed description of the categories can be found in Oliveira *et al*. [35].

#### Comparisons with other scores

The results of the maxZ score were compared with those of the MD and IC. Figures 2 and 3 show the MD and IC scores together with the -log(*p*)-values of the maxZ scores for the SH2 domain, peptidase M13, subtilase and *β*-lactamase family sequences. Scaling of the -log(*p*)-values was performed using zero as a minimum. The maximum value was obtained by calculating the -log(p)-values for each possible invariant position and defining the 5% percentile value to be the maximum. Blosum62 was used as a scoring matrix in the maxZ score. The default multiple sequence alignment parameters of ClustalX were used to calculate the MD score.

**SH2 domain** SH2 domains are binding modules recognizing phosphotyrosines and surrounding residues in polypeptides and proteins [39,40]. Many SH2 domains recognize especially residues +1 and +3 following the phosphotyrosine and form binding pockets for these amino acids [41]. All known SH2 domains share the same architecture, consisting of a central antiparallel *β*-sheet flanked by two *α*-helices. The central *β*-sheet (strands B, C and D) forms the core of the structure and includes most of the conserved residues.

All scores consider the positions forming the binding pocket as highly conserved (> 0.4). These include invariant *βB*5, which interacts with phosphotyrosine, and *βD*4 and *αA*2 (data not shown), which form the binding pocket for the phosphotyrosine [42] (Figure 2ab). Position *βD*6, which is also involved in forming the binding pocket, obtains lower conservation score values (≈ 0.2) indicating moderate conservation. The binding pockets for phosphotyrosine-following residues are formed by the *αB*-helix, especially positions *αB*5 – 6 are involved in forming the hydrophobic core for residue +3 [43]. Positions *βB*2, *αB*9 and *βF*3 are occupied with aromatic residues. The MaxZ and IC scores determine these five positions as highly conserved, whereas the MD score (0.2 – 0.4) determines positions *αB*9, and *βF*3 as moderately conserved (Figure 2c). The binding site for ligand residue +1 includes positions *βD*3 and *βD*5 [42]. While the maxZ and IC scores determine position *βD*5 as moderately conserved, the MD score (< 0.2) rather considers that position as unconserved (Figure 2b).

**Peptidase family M13** Peptidase family M13, also known as neprilysin family, consists of type II integral transmembrane proteins with short N-terminal cytoplasmic domain, a hydrophobic transmembrane region, and a large ectodomain containing a active site [44]. Three conserved motifs characterize all known M13 endopeptidases (the numbers are Pfam alignment positions): I:^0^vNAfY^4^, II:^63^XXHEXXH- -XX^73^, III:^147^EXXXD^151 ^(Figures 3abc). Additionally IV:^217^HXXXXXR^223 ^is conserved in neprilysins (Figure 3d).

All measures scored as highly conserved the residues H65, H69, E147 which are ligands for *Zn*^2+^, and E66 and H217, which are involved in catalysis (Figure 3bcd). The maxZ score values varied from 0.68 to 1 in the invariant positions occupied with different amino acids, whereas the corresponding MD score values were more stable. This was due to different diagonal values of the scoring matrix. The similar behavior was found in the position 219 of the motif IV, where proline was the most frequent residue. The maxZ score determined that position as highly conserved (0.84), whereas the other scores only considered it as moderately conserved (0.38 and 0.52). For the other important side-chains of N1, A2, D215, H217 and R223, which have a role in substrate binding, the behavior of the three scores was mostly very similar (Figure 3ad). The only exception was the position D215, which was considered as moderately conserved by the maxZ and IC scores (0.22 and 0.44), while the MD score considered it as unconserved. Another difference between the scores was in the positions 70 and 71 of the motif II, where the IC score could not determine these positions as inserts, but obtained considerably high conservation score values.

**Subtilisins** Pfam subtilase is a family of serine proteases consisting of S8 and S53 peptidase families of the MEROPS database. The S8 peptidases are divided into two subfamilies: S8A (e.g. subtilisin) and S8B (e.g. kexin). The sequences in the S8 family have a catalytic triad Asp/His/Ser. In the subfamily S8A, the active site residues occur (in the Pfam alignments) in the motifs I:^28^D-T/SG^31^, II:^254^HGTH^257 ^and III:^687^GTSMAXP^693^, and in the subfamily S8B in the motifs I:^28^D-DG^31^, II:^254^HGTR^257^, III:^687^GTSA/VA/SXP^693 ^(Figure 3efg).

All positions of the catalytic triad Asp/His/Ser were considered as highly conserved by each of the conservation scores. In the first motif, the maxZ and MD scores obtained high conservation score values (0.70–0.90) for the aspartic acid and glycine residues (Figure 3e). The middle position had three possible side-chains in the first motif, and hence, all scores determined that position as moderately conserved (0.34–0.42). In the second motif, there were more differences between the conservation scores: the maxZ score determined all the positions as highly conserved (0.71–1), the MD score determined the first three positions as highly conserved (0.74–1), whereas the fourth position obtained much lower score (0.31) (Figure 3f). The IC score determined only the first position as highly conserved (0.84), whereas the other positions obtained a slightly lower (0.49–0.63) conservation score values. Hence, only the maxZ score considered the whole motif as highly conserved. The MD score, on the contrary, obtained rather low conservation score values for the position 257, where subgroups S8A and S8B are conserved in different amino acids. The third motif was a good example of the behavior of the different scores in the invariant positions (Figure 3g). While the MD score obtained the highest score value 1 in all the invariant positions, the maxZ and IC scores were dependent on the side-chain. Nevertheless, the maxZ score determined all the invariant positions as highly conserved (0.68–1), whereas the IC score obtained somewhat lower scorings (0.49–0.73).

***β*-Lactamases ***β*-Lactamase family of Pfam contains sequences from many different groups including D-alanyl-D-alanine carboxypeptidase B, aminopeptidase, alkaline D-peptidase, animal D-Ala-D-Ala carboxypeptidase homologues, the class A and C *β*-lactamases and eukaryotic *β*-lactamase homologs. The family is very diverse outside the SXXK motif, S being the active side residue. For the sequences belonging to the S12 peptidase (D-Ala-D-Ala carboxypeptidase B) family in the MEROPS database, the active site motif is I:^120^SXTK^123^. It also has another motif: II:^306^YXN^308 ^(Figure 3hi).

All the scores determined the active site serine residue as highly conserved and provided a very similar conservation profile for the first motif (Figure 3h). In the second motif, the maxZ and IC score correctly determined the highly conserved position 306 with tyrosine/serine residues and considered the other residues as moderately conserved, while the MD score, on the contrary, failed to detect the highly conserved position 306, where it gave only 0.21 as a score value for that position (Figure 3i).

These results on the example families suggest that there are three main differences between the maxZ, MD and IC conservation scores. Firstly, since the maxZ score is strongly affected by the diagonal value of the scoring matrix used, it obtains slightly lower values for the positions occupied with very frequently occurring amino acids and slightly higher value for more rarely occurring amino acids than the other scores. For very frequently occurring amino acids, see for example position *αB*5 of the SH2 domain (Figure 2c) with highly conserved leucine or positions III:G687 and III:S689 of the subtilase family (Figure 3g), which obtain a much lower maxZ than MD score. In the opposite case, at positions *αB*8 – 9 of the SH2 domain, the high diagonal values of the scoring matrix for histidine and tryptophan offer a much more reliable scoring than the MD score (Figure 2c). Similarly, the result of the maxZ score for the position 11:306 of the *β*-lactamase family indicates that the position may be functionally important, whereas the result of the MC score indicates the contrary (Figure 3i). The maxZ score also determines different values for invariant positions with different amino acids, whereas MD score always gives the score of 1 for invariant positions. Secondly, as the maxZ score is entirely determined by the residue obtaining the greatest Z-score value, it is not affected by the other residues whose proportions may be very low, but a single conserved residue can already define a position as conserved (see position *βF*3 in Figure 2c or position S/Y306 in Figure 3i). Hence, the maxZ score may find important positions of the alignment, which were not found by the other scores. Thirdly, the maxZ and MD scores also consider gaps resulting in zero or very low scores for the insert positions of the alignment. The IC score, on the other hand, fails to detect the insert positions.

Taken together, all three scores behaved in a rather similar manner. The IC score does not take into account gaps, and thus its use is relevant only when the alignment does not include gaps. The maxZ and MD scores differ in some positions, which generally depend on the similarity matrix or grouping used with the maxZ score. For the Ras-like proteins, the maxZ score with groups of amino acids distinguishes slightly better than the other scores both the moderately conserved positions (Boxes 12–23) from the highly conserved positions (Box 11) and unconserved ones (Box 33). However, the results of Table 1 cannot be used to evaluate the IC score since entropy was used in the classification. For the SH2 domains, on the one hand, the maxZ score determines the positions forming the binding pocket for the phosphotyrosine and surrounding molecules mostly as highly conserved, but on the other hand, it also correctly determines the more variable loops between the *α*-helices and *β*-stands as unconserved. The MD and IC scores also perform well, but sometimes the MD score fails to detect the important positions, and the IC score is not capable in detecting the loops between the conserved structures.

#### Consensus sequence

As a by-product, the maxZ score also produces the consensus sequence for the multiple sequence alignment. According to formula (8), the consensus residue at each alignment position is defined as the residue with the greatest Z-score value. The legend of Figure 2 shows the consensus sequence for the part of the SH2 domain.

### Evaluating the AQ score for alignment quality

In this section, we evaluate the output of the alignment programs using alignment quality (AQ) based on the maxZ score and compare it to the sum of pairs (SP) and the column score (CS) quality scores [1]. First, we study the relationship between the individual AQ and SP scores. Then we compare the quality scores of 7 alignment methods using BAliBASE database [45]. Since the divergence from the reference values was substantially constant over different false discovery rate (FDR) values, the results are presented at FDR = 0.05.

#### Comparison of individual AQ and SP scores

**Figure 4:**
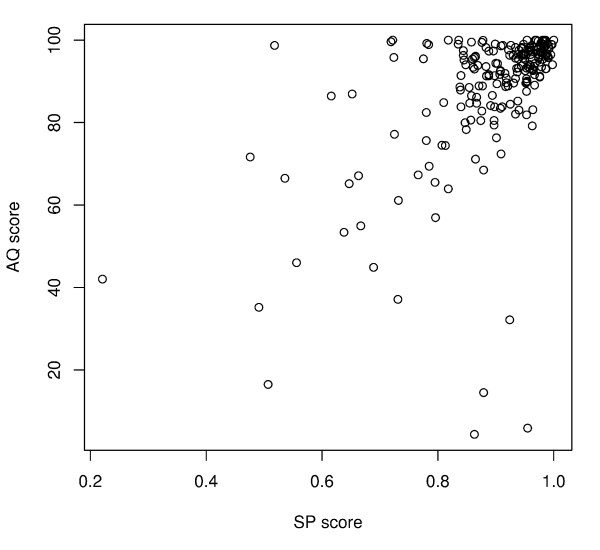
**Scatterplot between the AQ and SP scores for the Mafft (L-INS-i) alignments (r = 0.53)**. Four outlying alignments on the bottom right corner are from the reference sets 11 and 40.

We build 7 test alignments for each set of sequences in the BAliBASE database and compared the results of the AQ and SP scores. Figure 4 shows the scatterplot between the AQ and SP scores for the Mafft alignments (L-INS-i strategy) in different reference sets. The Spearman rank correlation coefficient between the AQ and SP scores was 0.53 for the L-INS-i alignments. The range of the correlation coefficient in the 7 alignments was from 0.53 to 0.67. Figure 4 shows a clear relationship between the quality scores. The three of the four outlying alignments on the lower right corner of Figure 4 are from the reference set 40. In these alignments, the SP scores also dramatically differed from the Column score values (CS = 0 in these alignments).

#### Alignment quality assessment

**Figure 5:**
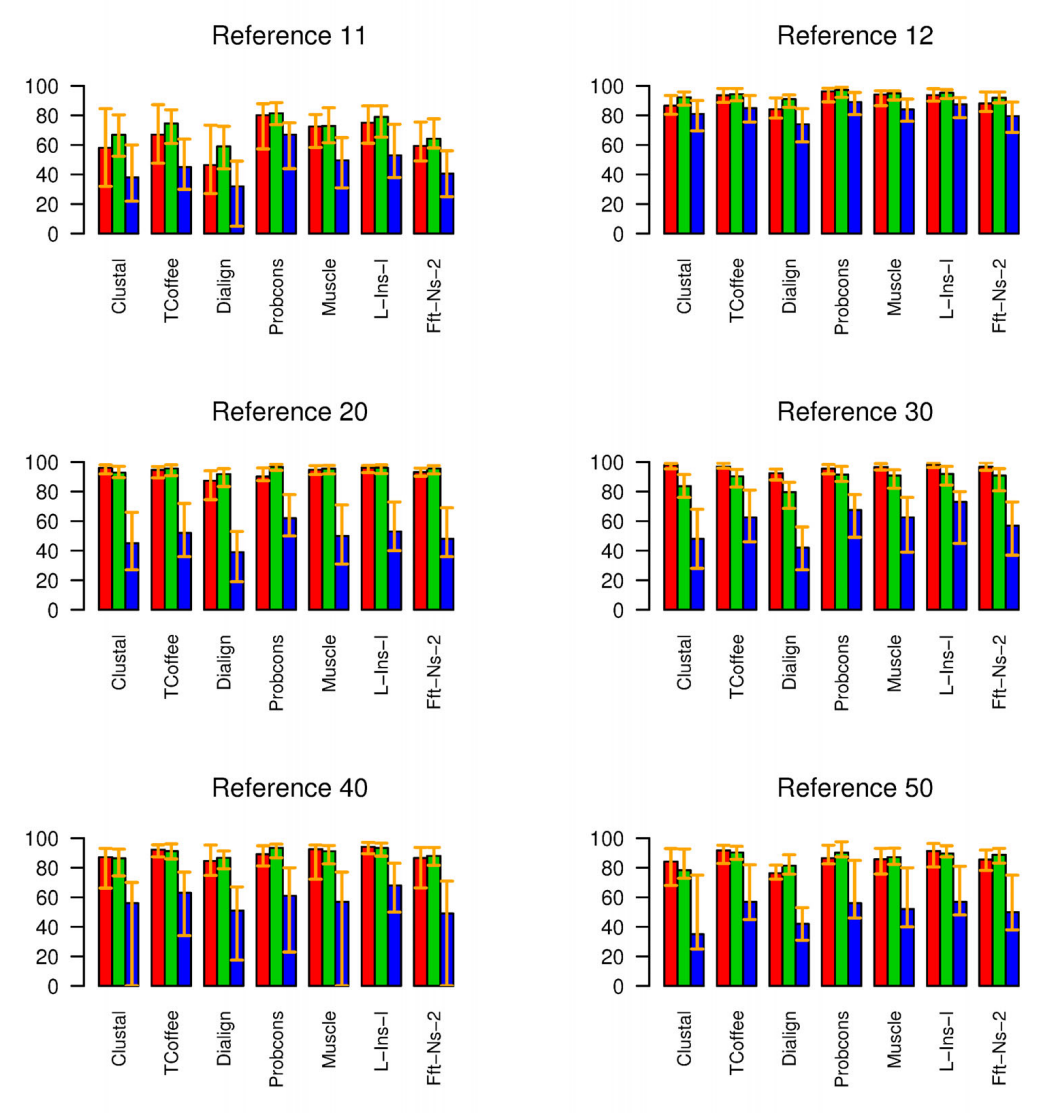
**Barplots for the median (red) AQ, (green) SP and (blue) CP scores in the BAliBASE reference sets**. Error bars show the 25% and 75% percentile values.

We compared the performance of the 7 alignment programs using five reference sets of the BAliBASE database. The first two reference sets of the BAliBASE include equi-distant sequences whose identity is less than 20% (ref 11) or between 20 and 40% (ref 12). According to the AQ score, the results on the reference set 11 indicate that Probcons was the best method aligning on the average 80% of the conserved residues correctly (Figure 5). The L-INS-i strategy of Mafft and Muscle also performed well obtaining quality scores only 5–7% lower than that of the Probcons. In the reference set 12, all the tested programs performed rather well (Figure 5). The Probcons, Muscle, L-INS-i and TCoffee obtained the highest alignment quality score values (94–96%). These methods did not differ from each other, but they differed from all the other methods (Table 2). The quality score was the worst in the alignments produced by Dialign, Clustal, or FFT-NS-2 strategy of Mafft showing 41–54% (ref 11) and 12–16% (ref 12) divergence from the reference alignment. The result of the SP score was very similar. The only relevant difference was the Probcons showing significant difference to the other programs in the both reference sets 11 and 12, even if the absolute difference between the methods was very low: the SP score of the Probcons and L-INS-i, for instance, differed from each other only 2%. The absolute CS scores were in all programs approximately 20% (ref 11) and 10% (ref 12) lower than that of the AQ and SP scores. In the reference set 12, the Probcons differed significantly from the other methods. In the reference set 11, the Probcons showed significant difference from all the other programs except the L-INS-i.

The aim of the reference set 20 is to test the ability of programs to align the sequence families having disrupted by an "orphan" sequence. The reference set 30 consists of subgroups of sequences whose residue identities between the subgroups are less than 25%. According to the AQ and SP measures, the quality of all alignments was very high in the reference sets 20 and 30 (Figure 5). In the reference set 20, the median scores varied from 87% to 96% and from 92% to 97% in the AQ and SP scorings, respectively, whereas the CS score obtained clearly lower scores varying from 39% to 62%. In the reference set 30, the overall SP (80–92%) and especially CP scoring (42–73%) was somewhat lower than that of the AQ scoring (92–98%). In the AQ scoring, the L-INS-i and Clustal were slightly better than the other methods aligning 96/98% (ref 20/ref 30) of the conserved residues correctly. The Muscle and TCoffee scored almost as well and did not differ significantly from the L-INS-i and Clustal (Table 2). In the reference set 30, additionally, the FFT-NS-2 and Probcons did not differ from the best scoring methods. The Dialign obtained clearly the lowest quality scores (87% in ref 20 and 92% in ref 30), and differed significantly from the other methods. In the reference set 20, the SP scoring of the Probcons showed significantly better performance than the other programs. The SP scores of the four best programs: Probcons, L-INS-i, TCoffee and Muscle, were, however, within the 1.2% range from each other. Another difference between the AQ and the other scores was that while with the AQ scoring the Clustal (96%) was among the four top methods and the Probcons (90%) was the second worst method, with the SP and CS scoring the Probcons obtained the best results (97%/62%) and the Clustal (93%/45%) was the second worse method differing significantly from the better scoring methods (Table 2).

**Table 2:** The alignment programs which obtained the highest AQ, SP and CS scores in different reference sets.

	Top Programs		
Reference set	AQ	SP	CS
11	Probcons, L-INS-i, Muscle	ProbCons	ProbCons, L-INS-i
12	Probcons, Muscle, L-INS-i, Tcoffee	ProbCons	ProbCons
20	L-INS-i, Clustal, Muscle, Tcoffee	ProbCons	ProbCons, L-INS-i
30	L-INS-i, Clustal, TCoffee, FFT-NS-2, Muscle, Probcons	L-INS-i, Probcons, Muscle, TCoffee	L-INS-i, Probcons, TCoffee, Muscle
40	L-INS-i, TCoffee	L-INS-i, Probcons, TCoffee	L-INS-i, TCoffee, Probcons
50	L-INS-i, TCoffee, Probcons, Muscle, FFT-NS-2, Clustal	TCoffee, Probcons, L-INS-i	L-INS-i, TCoffee, Probcons, Muscle

The programs have no statistically significant differences between each other in the particular set. Statistical analyses were performed using Wilcoxon signed rank test.

The reference set 40 contains sequences with N/C-terminal extensions. In this reference set, the median AQ and SP scores varied from 85% to 94% and the median CP score from 49% to 68%. The L-INS-i obtained the best AQ scores aligning 94% of the conserved residues correctly (Figure 5). The differences between the L-INS-i and the other methods, except the TCoffee, were statistically significant (Table 2). The performance of the three quality scores were very similar; the only difference was that with the SP and CS scorings the quality of the Probcons alignments were comparable with the quality of the L-INS-i and TCoffee alignments.

In the last reference set, the alignment includes sequences with internal insertions. In this reference set, the L-INS-i and TCoffee obtained approximately 5 to 6% better results than the other methods aligning more than 91% of the conserved residues correctly when the AQ scoring was used (Figure 5). The differences were, however, statistically significant only with respect to the Dialign (Table 2). According to the SP score, the TCoffee, Probcons and L-INS-i differentiated between the lower scoring methods FFT-NS-2, Muscle, Dialign and Clustal, even if the differences in the median values were very low. In the CP score, the result was similar to that of the SP score. The only difference was the Muscle, which ranked among the four best programs.

To summarize, in the BAliBASE database the L-INS-i, Probcons, Muscle, TCoffee and Clustal all produced alignments with very high quality, whereas the FFT-NS-2 and Dialign performed generally worse than the other methods. The overall best method was the L-INS-i which was among the significantly best methods in all six reference sets (Table 2). The Probcons performed best in the reference sets 11 and 12, whereas in the other sets, the L-INS-i was the best scoring method. In the SP score, the Probcons differed significantly from the other methods in the reference sets 11–20 and was among the best scoring methods in all reference sets. The CS score results in much lower values than the other scores, but the ranking of the methods was very near to that of the SP score. In both scores, the Probcons, L-INS-, TCoffee and in some references Muscle produced the best alignments, whereas the Clustal, FFT-NS-2 and Dialign performed worse.

## Discussion

In this paper, we have introduced a novel approach to objective alignment quality scoring. Unlike most of the existing methods, the proposed AQ score is not heuristic but is based on statistical theory. The score is mathematically motivated and its asymptotic properties are well known. The AQ score does not handle all alignment positions equally but concentrates on conserved positions only. In the present work, the AQ score is calculated with respect to the reference alignment. The future aim is to use the conserved alignment positions without the reference alignment. The proportion of conserved residues *ConsAA *can be used to assess the quality of the alignments also when the reference alignment is not available. Our preliminary results show a strong correlation between the predicted and reference alignment based AQ score values (data not shown here).

The proposed scoring method is based on integrating the statistical hypotheses testing methods into the profile analysis framework. The attraction of profile analysis lies in the convenient treatment of the symbol frequency vector, which allows not only the incorporation of any classification or symbol similarity matrix but also the possibility to consider the influence of gaps and weights in a very simple manner [6]. Hence a score based on profile analysis immediately fulfills the six criteria set as requirements of a good conservation score by Valdar [17]. A drawback of the current maxZ score is that the sequence weighting is not taken into consideration. Weighting the profiles with an appropriate value would allow the evolutionary relationships of the sequences in the multiple sequence alignment to be considered.

The AQ score is based on comparing the number of conserved alignment positions between the test and the reference alignments, as assessed with the maxZ score, so that the dependency between the alignment positions is also considered. The multiple comparison problem is handled by using false discovery rate when choosing the conserved positions. To estimate the significance of the observed maxZ scores, we used the IS method. In genetics applications, the IS method has previously been successfully applied to the binomial distribution [46,47]. The application of the IS method to multinomial distribution is, however, not a trivial task because the parameter space is multidimensional. We used a mixture distribution as a sampling distribution for the multinomial distribution. With the help of simulations, we sought for the appropriate parameter values of the mixture distribution and approximated the number of the samples needed for the proper estimation of the significance values (see Additional file 10).

In addition to developing an alignment quality scoring framework, our second objective was to test the alignment quality of commonly used multiple sequences alignment programs. We evaluated the quality of 7 alignment methods. The overall performance of the L-INS-i strategy of Mafft was the best. The Probcons worked best with groups of equi-distant sequences having residue identity less than 20% (ref 11) or 20–40% (ref 12). The L-INS-i and Clustal performed best with the reference set that consists of families aligned with a highly divergent "orphan" sequence (ref 20) or groups of equi-distant distantly related sequences (ref 30). The L-INS-i and TCoffee worked best when the sequences contained N/C-terminal extensions (ref 40) or internal insertions (ref 50). It should be noted, however, that the differences between the most of the alignment methods were negligible; in addition to the L-INS-i, also the Probcons, Muscle, TCoffee and Clustal produced alignments with very high quality. The Dialign and FFT-NS-2 strategy of Mafft, on the contrary, performed clearly worse than the other methods. The comparison between the Mafft and Muscle was potentially biased because the Muscle was run using default settings. Running the Muscle with the most accurate options would probably have affected the results.

We evaluated the quality of the alignment software using the BAliBASE 3 database. Previous studies using the BAliBASE database have been performed for the database version 2 [12,13,3,10,1]. The drawback of that database is that some of the reference alignments consist of a few sequences only. In the version 3, the reference sets have more sequences and therefore the current database suits better for statistical scoring of the alignment quality. The results are rather similar to those obtained in the previous studies using the SP or CP scores or modified versions of them [12,13,3,10,1]. In our study, the performance of Mafft is better than reported earlier. This is because the previous results have been obtained using the NW-NS-i strategy, whereas we used the L-INS-i, the most accurate strategy of Mafft at the moment (see Mafft web page for more details [48]). Another difference was in the performance of Probcons: Do *et al*. [12] showed that in the reference sets 20–50, Probcons outperformed the other methods, while with our AQ scoring, the performance of the Probcons was poorer than that of the L-INS-i and TCoffee.

## Conclusion

We have presented a statistical approach to alignment quality scoring. The quality is characterized on the basis of conserved position information only, which is defined by using the modified Z score in conjunction with the profile analysis framework. The significance tests based on the importance sampling method define the conserved positions and false discovery rate correct the error caused by multiple testing. The final AQ score accounts for the residue frequency over the conserved alignment positions.

We have compared the AQ scores of the 7 alignment methods using the BAliBASE as a benchmarking database. The results indicates that even if the L-INS-i obtained the best overall result, there are no great differences between the best scoring alignment methods: L-INS-i, Probcons, Muscle, TCoffee and Clustal whereas the FFT-NS-2 and Dialign usually scored worse. The comparison of the AQ and SP scores gave similar results indicating that the AQ score is a reliable method for assessing the quality of the multiple sequence alignments.

## Methods

### The maximum Z-score

Let us assume that the occurrences of symbols at each alignment position are sampled from a discrete distribution with *β*_1_, *β*_2_, ..., *β*_*J *_as the true symbol probabilities. For DNA sequences *J *= 4 (bases A, C, G, and T), and for protein sequences *J *= 20 (amino acids A, C,..., Y). The statistical properties of the alignment are then completely characterized by the multinomial distribution model. In particular, the probability of a position with observed symbol frequencies *n*_1_, *n*_2_, ..., *n*_*J *_is proportional to the product:

ℙ(n1,n2,...,nJ|β1,β2,...,βJ)~∏j=1Jβjnj.     (1)
 MathType@MTEF@5@5@+=feaafiart1ev1aaatCvAUfKttLearuWrP9MDH5MBPbIqV92AaeXatLxBI9gBaebbnrfifHhDYfgasaacH8akY=wiFfYdH8Gipec8Eeeu0xXdbba9frFj0=OqFfea0dXdd9vqai=hGuQ8kuc9pgc9s8qqaq=dirpe0xb9q8qiLsFr0=vr0=vr0dc8meaabaqaciaacaGaaeqabaqabeGadaaakeaatuuDJXwAK1uy0HMmaeHbfv3ySLgzG0uy0HgiuD3BaGabaiab=LriqjabcIcaOiabd6gaUnaaBaaaleaacqaIXaqmaeqaaOGaeiilaWIaemOBa42aaSbaaSqaaiabikdaYaqabaGccqGGSaalcqGGUaGlcqGGUaGlcqGGUaGlcqGGSaalcqWGUbGBdaWgaaWcbaGaemOsaOeabeaakiabcYha8HGaciab+j7aInaaBaaaleaacqaIXaqmaeqaaOGaeiilaWIae4NSdi2aaSbaaSqaaiabikdaYaqabaGccqGGSaalcqGGUaGlcqGGUaGlcqGGUaGlcqGGSaalcqGFYoGydaWgaaWcbaGaemOsaOeabeaakiabcMcaPiabc6ha+naarahabaGae4NSdi2aa0baaSqaaiabdQgaQbqaaiabd6gaUnaaBaaameaacqWGQbGAaeqaaaaaaSqaaiabdQgaQjabg2da9iabigdaXaqaaiabdQeakbqdcqGHpis1aOGaeiOla4IaaCzcaiaaxMaadaqadaqaaiabigdaXaGaayjkaiaawMcaaaaa@6859@

The probability vector ***β ***= (*β*_1_, *β*_2_, ..., *β*_*J*_) must satisfy the stochastic constraints: *β*_*j *_≥ 0 and ∑j=1Jβj=1
 MathType@MTEF@5@5@+=feaafiart1ev1aaatCvAUfKttLearuWrP9MDH5MBPbIqV92AaeXatLxBI9gBaebbnrfifHhDYfgasaacH8akY=wiFfYdH8Gipec8Eeeu0xXdbba9frFj0=OqFfea0dXdd9vqai=hGuQ8kuc9pgc9s8qqaq=dirpe0xb9q8qiLsFr0=vr0=vr0dc8meaabaqaciaacaGaaeqabaqabeGadaaakeaadaaeWaqaaGGaciab=j7aInaaBaaaleaacqWGQbGAaeqaaaqaaiabdQgaQjabg2da9iabigdaXaqaaiabdQeakbqdcqGHris5aOGaeyypa0JaeGymaedaaa@3844@. Let *N *be the number of sequences in the alignment and n=∑i=1Jnj
 MathType@MTEF@5@5@+=feaafiart1ev1aaatCvAUfKttLearuWrP9MDH5MBPbIqV92AaeXatLxBI9gBaebbnrfifHhDYfgasaacH8akY=wiFfYdH8Gipec8Eeeu0xXdbba9frFj0=OqFfea0dXdd9vqai=hGuQ8kuc9pgc9s8qqaq=dirpe0xb9q8qiLsFr0=vr0=vr0dc8meaabaqaciaacaGaaeqabaqabeGadaaakeaacqWGUbGBcqGH9aqpdaaeWaqaaiabd6gaUnaaBaaaleaacqWGQbGAaeqaaaqaaiabdMgaPjabg2da9iabigdaXaqaaiabdQeakbqdcqGHris5aaaa@386A@ the actual number of symbols observed at the position, that is, the number of gaps subtracted from *N*. By maximizing the likelihood function (1) subject to the stochastic constraints, it can be easily shown that the maximum likelihood (ML) estimator ***b ***of the vector ***β ***is given in the form of the observed relative frequencies

bj=β^jML=njn,j=1,2,...,J.     (2)
 MathType@MTEF@5@5@+=feaafiart1ev1aaatCvAUfKttLearuWrP9MDH5MBPbIqV92AaeXatLxBI9gBaebbnrfifHhDYfgasaacH8akY=wiFfYdH8Gipec8Eeeu0xXdbba9frFj0=OqFfea0dXdd9vqai=hGuQ8kuc9pgc9s8qqaq=dirpe0xb9q8qiLsFr0=vr0=vr0dc8meaabaqaciaacaGaaeqabaqabeGadaaakeaafaqabeqacaaabaGaemOyai2aaSbaaSqaaiabdQgaQbqabaGccqGH9aqpiiGacuWFYoGygaqcamaaDaaaleaacqWGQbGAaeaacqqGnbqtcqqGmbataaGccqGH9aqpdaWcaaqaaiabd6gaUnaaBaaaleaacqWGQbGAaeqaaaGcbaGaemOBa4gaaiabcYcaSaqaaiabdQgaQjabg2da9iabigdaXiabcYcaSiabikdaYiabcYcaSiabc6caUiabc6caUiabc6caUiabcYcaSiabdQeakbaacqGGUaGlcaWLjaGaaCzcamaabmaabaGaeGOmaidacaGLOaGaayzkaaaaaa@4BCF@

According to the properties of multinomial distribution, the expectation vector and the covariance matrix of the estimate are ***β ***and **Σ**, respectively, where

∑ij=βi(δij−βj)n,i,j=1,2,...,J.     (3)
 MathType@MTEF@5@5@+=feaafiart1ev1aaatCvAUfKttLearuWrP9MDH5MBPbIqV92AaeXatLxBI9gBaebbnrfifHhDYfgasaacH8akY=wiFfYdH8Gipec8Eeeu0xXdbba9frFj0=OqFfea0dXdd9vqai=hGuQ8kuc9pgc9s8qqaq=dirpe0xb9q8qiLsFr0=vr0=vr0dc8meaabaqaciaacaGaaeqabaqabeGadaaakeaafaqabeqacaaabaWaaabeaeaacqGH9aqpdaWcaaqaaGGaciab=j7aInaaBaaaleaacqWGPbqAaeqaaOGaeiikaGIae8hTdq2aaSbaaSqaaiabdMgaPjabdQgaQbqabaGccqGHsislcqWFYoGydaWgaaWcbaGaemOAaOgabeaakiabcMcaPaqaaiabd6gaUbaaaSqaaiabdMgaPjabdQgaQbqab0GaeyyeIuoakiabcYcaSaqaaiabdMgaPjabcYcaSiabdQgaQjabg2da9iabigdaXiabcYcaSiabikdaYiabcYcaSiabc6caUiabc6caUiabc6caUiabcYcaSiabdQeakjabc6caUaaacaWLjaGaaCzcamaabmaabaGaeG4mamdacaGLOaGaayzkaaaaaa@53DC@

The Kronecker's delta function is defined by *δ*_*jj *_= 1 and *δ*_*ij *_= 0 for all *i *≠ *j*.

Given an appropriate symbol similarity matrix ***C***, the entries of the profile ***f ***= ***Cb ***are expressed as linear combinations

fi=∑j=1Jbjcij=ciTb,     (4)
 MathType@MTEF@5@5@+=feaafiart1ev1aaatCvAUfKttLearuWrP9MDH5MBPbIqV92AaeXatLxBI9gBaebbnrfifHhDYfgasaacH8akY=wiFfYdH8Gipec8Eeeu0xXdbba9frFj0=OqFfea0dXdd9vqai=hGuQ8kuc9pgc9s8qqaq=dirpe0xb9q8qiLsFr0=vr0=vr0dc8meaabaqaciaacaGaaeqabaqabeGadaaakeaacqWGMbGzdaWgaaWcbaGaemyAaKgabeaakiabg2da9maaqahabaGaemOyai2aaSbaaSqaaiabdQgaQbqabaGccqWGJbWydaWgaaWcbaGaemyAaKMaemOAaOgabeaaaeaacqWGQbGAcqGH9aqpcqaIXaqmaeaacqWGkbGsa0GaeyyeIuoakiabg2da9Gqadiab=ngaJnaaDaaaleaacqWGPbqAaeaacqWGubavaaGccqWFIbGycqGGSaalcaWLjaGaaCzcamaabmaabaGaeGinaqdacaGLOaGaayzkaaaaaa@4968@

where the vector ci=(cij)j=1J
 MathType@MTEF@5@5@+=feaafiart1ev1aaatCvAUfKttLearuWrP9MDH5MBPbIqV92AaeXatLxBI9gBaebbnrfifHhDYfgasaacH8akY=wiFfYdH8Gipec8Eeeu0xXdbba9frFj0=OqFfea0dXdd9vqai=hGuQ8kuc9pgc9s8qqaq=dirpe0xb9q8qiLsFr0=vr0=vr0dc8meaabaqaciaacaGaaeqabaqabeGadaaakeaaieWacqWFJbWydaWgaaWcbaGaemyAaKgabeaakiabg2da9iabcIcaOiabdogaJnaaBaaaleaacqWGPbqAcqWGQbGAaeqaaOGaeiykaKYaa0baaSqaaiabdQgaQjabg2da9iabigdaXaqaaiabdQeakbaaaaa@3B26@ denotes the *i*^th ^row of ***C ***related to the symbols *i *= 1, 2, ..., *J*. The degree of positional conservation is calculated with respect to a predefined background distribution ***β***^0 ^= (β10,β20,...,βJ0
 MathType@MTEF@5@5@+=feaafiart1ev1aaatCvAUfKttLearuWrP9MDH5MBPbIqV92AaeXatLxBI9gBaebbnrfifHhDYfgasaacH8akY=wiFfYdH8Gipec8Eeeu0xXdbba9frFj0=OqFfea0dXdd9vqai=hGuQ8kuc9pgc9s8qqaq=dirpe0xb9q8qiLsFr0=vr0=vr0dc8meaabaqaciaacaGaaeqabaqabeGadaaakeaaiiGacqWFYoGydaqhaaWcbaGaeGymaedabaGaeGimaadaaOGaeiilaWIae8NSdi2aa0baaSqaaiabikdaYaqaaiabicdaWaaakiabcYcaSiabc6caUiabc6caUiabc6caUiabcYcaSiab=j7aInaaDaaaleaacqWGkbGsaeaacqaIWaamaaaaaa@3D3C@) under the null hypothesis H_0 _: ***β ***= ***β***^0^. The theoretical expectation vector and covariance matrix of the profile under H_0 _are

E(f)=Cβ0andℂov(f)=CΣ0CT,     (5)
 MathType@MTEF@5@5@+=feaafiart1ev1aaatCvAUfKttLearuWrP9MDH5MBPbIqV92AaeXatLxBI9gBaebbnrfifHhDYfgasaacH8akY=wiFfYdH8Gipec8Eeeu0xXdbba9frFj0=OqFfea0dXdd9vqai=hGuQ8kuc9pgc9s8qqaq=dirpe0xb9q8qiLsFr0=vr0=vr0dc8meaabaqaciaacaGaaeqabaqabeGadaaakeaafaqabeqadaaabaWefv3ySLgznfgDOjdaryqr1ngBPrginfgDObcv39gaiqaacqWFecFrcqGGOaakieWacqGFMbGzcqGGPaqkcqGH9aqpcqGFdbWqiiGacqqFYoGydaahaaWcbeqaaiabicdaWaaaaOqaaiabbggaHjabb6gaUjabbsgaKbqaaiab=jqidjabb+gaVjabbAha2jabcIcaOiab+zgaMjabcMcaPiabg2da9iab+neadHGabiab8n6atnaaCaaaleqabaGaeGimaadaaOGae43qam0aaWbaaSqabeaacqWGubavaaaaaOGaeiilaWIaaCzcaiaaxMaadaqadaqaaiabiwda1aGaayjkaiaawMcaaaaa@5716@

where the entries of **Σ**^0 ^are defined as in (3) with *β*_*j *_replaced by βj0
 MathType@MTEF@5@5@+=feaafiart1ev1aaatCvAUfKttLearuWrP9MDH5MBPbIqV92AaeXatLxBI9gBaebbnrfifHhDYfgasaacH8akY=wiFfYdH8Gipec8Eeeu0xXdbba9frFj0=OqFfea0dXdd9vqai=hGuQ8kuc9pgc9s8qqaq=dirpe0xb9q8qiLsFr0=vr0=vr0dc8meaabaqaciaacaGaaeqabaqabeGadaaakeaaiiGacqWFYoGydaqhaaWcbaGaemOAaOgabaGaeGimaadaaaaa@30CC@. After standardizing the individual profiles (4) with the corresponding quantities (5), the final *Z*-score takes the form

Zi=ciT(b−β0)ciTΣ0ci,i=1,2,...,J.     (6)
 MathType@MTEF@5@5@+=feaafiart1ev1aaatCvAUfKttLearuWrP9MDH5MBPbIqV92AaeXatLxBI9gBaebbnrfifHhDYfgasaacH8akY=wiFfYdH8Gipec8Eeeu0xXdbba9frFj0=OqFfea0dXdd9vqai=hGuQ8kuc9pgc9s8qqaq=dirpe0xb9q8qiLsFr0=vr0=vr0dc8meaabaqaciaacaGaaeqabaqabeGadaaakeaafaqabeqacaaabaGaemOwaO1aaSbaaSqaaiabdMgaPbqabaGccqGH9aqpdaWcaaqaaGqadiab=ngaJnaaDaaaleaacqWGPbqAaeaacqWGubavaaGccqGGOaakcqWFIbGycqGHsisliiGacqGFYoGydaahaaWcbeqaaiabicdaWaaakiabcMcaPaqaamaakaaabaGae83yam2aa0baaSqaaiabdMgaPbqaaiabdsfaubaaiiqakiab9n6atnaaCaaaleqabaGaeGimaadaaOGae83yam2aaSbaaSqaaiabdMgaPbqabaaabeaaaaGccqGGSaalaeaacqWGPbqAcqGH9aqpcqaIXaqmcqGGSaalcqaIYaGmcqGGSaalcqGGUaGlcqGGUaGlcqGGUaGlcqGGSaalcqWGkbGscqGGUaGlaaGaaCzcaiaaxMaadaqadaqaaiabiAda2aGaayjkaiaawMcaaaaa@5548@

The statistic maps the residues of an alignment position onto a real number according to the observed symbol frequencies and their similarities among the symbol classes. If we use binary symbol similarities ci=(δij)j=120
 MathType@MTEF@5@5@+=feaafiart1ev1aaatCvAUfKttLearuWrP9MDH5MBPbIqV92AaeXatLxBI9gBaebbnrfifHhDYfgasaacH8akY=wiFfYdH8Gipec8Eeeu0xXdbba9frFj0=OqFfea0dXdd9vqai=hGuQ8kuc9pgc9s8qqaq=dirpe0xb9q8qiLsFr0=vr0=vr0dc8meaabaqaciaacaGaaeqabaqabeGadaaakeaaieWacqWFJbWydaWgaaWcbaGaemyAaKgabeaakiabg2da9iabcIcaOGGaciab+r7aKnaaBaaaleaacqWGPbqAcqWGQbGAaeqaaOGaeiykaKYaa0baaSqaaiabdQgaQjabg2da9iabigdaXaqaaiabikdaYiabicdaWaaaaaa@3C45@, we obtain a special case of the *Z*-score where the similarities among the symbols are ignored. Generally, ***c***_*i *_can have any fixed form appropriate for the study. In the present application, the score we propose for the positional conservation analysis is obtained by selecting the maximal *Z*_*i*_-value over the symbol classes *i *= 1, 2, ..., *J*. We call this statistic the maximum *Z*-score and abbreviate it as maxZ:

max⁡Z=max⁡i=1,2,...,JZi.     (7)
 MathType@MTEF@5@5@+=feaafiart1ev1aaatCvAUfKttLearuWrP9MDH5MBPbIqV92AaeXatLxBI9gBaebbnrfifHhDYfgasaacH8akY=wiFfYdH8Gipec8Eeeu0xXdbba9frFj0=OqFfea0dXdd9vqai=hGuQ8kuc9pgc9s8qqaq=dirpe0xb9q8qiLsFr0=vr0=vr0dc8meaabaqaciaacaGaaeqabaqabeGadaaakeaacyGGTbqBcqGGHbqycqGG4baEcqqGAbGwcqGH9aqpdaWfqaqaaiGbc2gaTjabcggaHjabcIha4bWcbaGaemyAaKMaeyypa0JaeGymaeJaeiilaWIaeGOmaiJaeiilaWIaeiOla4IaeiOla4IaeiOla4IaeiilaWIaemOsaOeabeaakiabdQfaAnaaBaaaleaacqWGPbqAaeqaaOGaeiOla4IaaCzcaiaaxMaadaqadaqaaiabiEda3aGaayjkaiaawMcaaaaa@49A3@

We assume that a single conserved symbol class can already define the position as conserved. The symbol class obtaining the maximum *Z*-score, i.e.

Cons = argmax_*i *= 1,2,...,*J*_*Z*_*i *_    (8)

defines the consensus residue for the particular alignment position.

### The significance of the observed maxZ score

Once the maxZ-statistic has been evaluated, the next question concerns its significance, namely, whether the observed value of the maxZ-statistic is large enough to justify the rejection of H_0 _at a particular position. In this section, the problem of identifying conserved positions of a multiple alignment is considered as a statistical hypothesis testing problem. This consists of testing the null hypothesis H_0 _: *β*_*j *_= βj0
 MathType@MTEF@5@5@+=feaafiart1ev1aaatCvAUfKttLearuWrP9MDH5MBPbIqV92AaeXatLxBI9gBaebbnrfifHhDYfgasaacH8akY=wiFfYdH8Gipec8Eeeu0xXdbba9frFj0=OqFfea0dXdd9vqai=hGuQ8kuc9pgc9s8qqaq=dirpe0xb9q8qiLsFr0=vr0=vr0dc8meaabaqaciaacaGaaeqabaqabeGadaaakeaaiiGacqWFYoGydaqhaaWcbaGaemOAaOgabaGaeGimaadaaaaa@30CC@ against the alternative H_*A *_: *β*_*j *_≥ βj0
 MathType@MTEF@5@5@+=feaafiart1ev1aaatCvAUfKttLearuWrP9MDH5MBPbIqV92AaeXatLxBI9gBaebbnrfifHhDYfgasaacH8akY=wiFfYdH8Gipec8Eeeu0xXdbba9frFj0=OqFfea0dXdd9vqai=hGuQ8kuc9pgc9s8qqaq=dirpe0xb9q8qiLsFr0=vr0=vr0dc8meaabaqaciaacaGaaeqabaqabeGadaaakeaaiiGacqWFYoGydaqhaaWcbaGaemOAaOgabaGaeGimaadaaaaa@30CC@, *j *= 1, 2,... *J*, where at least one inequality is proper. The problems caused by multiple comparisons within the position can be avoided by using the maxZ-statistic (7) as a test statistic, instead of the individual Z_*i*_-scores (6). Our aim is to test whether the observed value of maxZ is significantly larger than that which would be likely to arise under H_0 _due to random variation. The significance *p *of the observed value maxZ is formally defined by the tail probability function ℙ(maxZ) = 1 - ℙ(maxZ|H_0_). The smaller the *p*-value, the more extreme the maxZ-statistic and the stronger the evidence against H_0_. When the exact null distribution ℙ(maxZ|H_0_) is not available, it is essential to have widely applicable procedures that provide good approximation. Two approaches to approximate the theoretical null distribution are described below.

**Monte Carlo **(MC) approximation is perhaps the most frequently used non-parametric method for estimating the significance of an observed test statistic [49]. In the MC method, the samples are generated from the background distribution, and the null distribution is approximated through the cumulative sample distribution function. In the other words, the significance of the maxZ score is obtained by calculating the proportion of samples whose maxZ score is greater or equal to the observed maxZ value. However, because of the 20 dimensional parameter space, even with very large sample sizes, the probability of obtaining such an observation is very near to zero. Therefore the MC procedure is very ineffective and results in zero *p*-values with alignment positions which are only moderately conserved.

**Importance sampling **(IS), also referred as the weighted bootstrap re-sampling method, is a variant of the ordinary MC method [7,50]. Let us denote by *y *= *n*_1_, *n*_2_, ... , *n*_*J *_the sample of symbol frequencies from the multinomial distribution *g*^0 ^under H_0 _and *y*_*obs *_the observed symbol frequencies at one alignment position. Define *t*(*y*) as an indicator function *t*(*y*) = I{max⁡Z(y)≥max⁡Z(yobs)}
 MathType@MTEF@5@5@+=feaafiart1ev1aaatCvAUfKttLearuWrP9MDH5MBPbIqV92AaeXatLxBI9gBaebbnrfifHhDYfgasaacH8akY=wiFfYdH8Gipec8Eeeu0xXdbba9frFj0=OqFfea0dXdd9vqai=hGuQ8kuc9pgc9s8qqaq=dirpe0xb9q8qiLsFr0=vr0=vr0dc8meaabaqaciaacaGaaeqabaqabeGadaaakeaacqWGjbqsdaWgaaWcbaGaei4EaSNagiyBa0MaeiyyaeMaeiiEaGNaeeOwaOLaeiikaGIaemyEaKNaeiykaKIaeyyzImRagiyBa0MaeiyyaeMaeiiEaGNaeeOwaOLaeiikaGIaemyEaK3aaSbaaWqaaiabd+gaVjabdkgaIjabdohaZbqabaWccqGGPaqkcqGG9bqFaeqaaaaa@4830@. The general idea of the importance sampling is to draw samples from any such distribution g* where all realizations which are possible in *g*^0 ^are also possible in g*. By choosing g*(*y*) ∝ *t*(*y*)*g*^*o*^(*y*) as an IS distribution and taking infinitely many samples from g*, the exact observed significance level is given by

ℙ(max⁡Z(y)≥max⁡Z(yobs))=∑i=0∞t(yi)go(yi)g*(yi)g*(yi).
 MathType@MTEF@5@5@+=feaafiart1ev1aaatCvAUfKttLearuWrP9MDH5MBPbIqV92AaeXatLxBI9gBaebbnrfifHhDYfgasaacH8akY=wiFfYdH8Gipec8Eeeu0xXdbba9frFj0=OqFfea0dXdd9vqai=hGuQ8kuc9pgc9s8qqaq=dirpe0xb9q8qiLsFr0=vr0=vr0dc8meaabaqaciaacaGaaeqabaqabeGadaaakeaatuuDJXwAK1uy0HMmaeHbfv3ySLgzG0uy0HgiuD3BaGabaiab=LriqjabcIcaOiGbc2gaTjabcggaHjabcIha4jabbQfaAjabcIcaOiabdMha5jabcMcaPiabgwMiZkGbc2gaTjabcggaHjabcIha4jabbQfaAjabcIcaOiabdMha5naaBaaaleaacqWGVbWBcqWGIbGycqWGZbWCaeqaaOGaeiykaKIaeiykaKIaeyypa0ZaaabCaeaadaWcaaqaaiabdsha0jabcIcaOiabdMha5naaBaaaleaacqWGPbqAaeqaaOGaeiykaKIaem4zaC2aaWbaaSqabeaacqWGVbWBaaGccqGGOaakcqWG5bqEdaWgaaWcbaGaemyAaKgabeaakiabcMcaPaqaaiabdEgaNjabcQcaQiabcIcaOiabdMha5naaBaaaleaacqWGPbqAaeqaaOGaeiykaKcaaaWcbaGaemyAaKMaeyypa0JaeGimaadabaGaeyOhIukaniabggHiLdGccqWGNbWzcqGGQaGkcqGGOaakcqWG5bqEdaWgaaWcbaGaemyAaKgabeaakiabcMcaPiabc6caUaaa@7514@

Since this is an expectation of t(y)go(y)g*(y)
 MathType@MTEF@5@5@+=feaafiart1ev1aaatCvAUfKttLearuWrP9MDH5MBPbIqV92AaeXatLxBI9gBaebbnrfifHhDYfgasaacH8akY=wiFfYdH8Gipec8Eeeu0xXdbba9frFj0=OqFfea0dXdd9vqai=hGuQ8kuc9pgc9s8qqaq=dirpe0xb9q8qiLsFr0=vr0=vr0dc8meaabaqaciaacaGaaeqabaqabeGadaaakeaacqWG0baDcqGGOaakcqWG5bqEcqGGPaqkdaWcaaqaaiabdEgaNnaaCaaaleqabaGaem4Ba8gaaOGaeiikaGIaemyEaKNaeiykaKcabaGaem4zaCMaeiOkaOIaeiikaGIaemyEaKNaeiykaKcaaaaa@3CDC@ with respect to g*, we can approximate the observed significance level by taking *K *samples from g* and calculating an empirical tail probability function

ℙIS(max⁡Z(y)≥max⁡Z(yobs))=1K∑i=1Kt(yi)go(yi)g*(yi).     (9)
 MathType@MTEF@5@5@+=feaafiart1ev1aaatCvAUfKttLearuWrP9MDH5MBPbIqV92AaeXatLxBI9gBaebbnrfifHhDYfgasaacH8akY=wiFfYdH8Gipec8Eeeu0xXdbba9frFj0=OqFfea0dXdd9vqai=hGuQ8kuc9pgc9s8qqaq=dirpe0xb9q8qiLsFr0=vr0=vr0dc8meaabaqaciaacaGaaeqabaqabeGadaaakeaatuuDJXwAK1uy0HMmaeHbfv3ySLgzG0uy0HgiuD3BaGabaiab=LriqnaaBaaaleaacqWGjbqscqWGtbWuaeqaaOGaeiikaGIagiyBa0MaeiyyaeMaeiiEaGNaeeOwaOLaeiikaGIaemyEaKNaeiykaKIaeyyzImRagiyBa0MaeiyyaeMaeiiEaGNaeeOwaOLaeiikaGIaemyEaK3aaSbaaSqaaiabd+gaVjabdkgaIjabdohaZbqabaGccqGGPaqkcqGGPaqkcqGH9aqpdaWcaaqaaiabigdaXaqaaiabdUealbaadaaeWbqaaiabdsha0jabcIcaOiabdMha5naaBaaaleaacqWGPbqAaeqaaOGaeiykaKYaaSaaaeaacqWGNbWzdaahaaWcbeqaaiabd+gaVbaakiabcIcaOiabdMha5naaBaaaleaacqWGPbqAaeqaaOGaeiykaKcabaGaem4zaCMaeiOkaOIaeiikaGIaemyEaK3aaSbaaSqaaiabdMgaPbqabaGccqGGPaqkaaaaleaacqWGPbqAcqGH9aqpcqaIXaqmaeaacqWGlbWsa0GaeyyeIuoakiabc6caUiaaxMaacaWLjaWaaeWaaeaacqaI5aqoaiaawIcacaGLPaaaaaa@763F@

This gives us an IS estimate of the observed significance level.

A possible drawback to using the simulated distribution is that the *p*-value can be zero in the highly conserved positions. Hence several highly conserved positions may obtain the same score, and they cannot be distinguished from each other. In order to avoid this, we used the following approximation for the significance value:

ℙ(max⁡Z(y)≥max⁡Z(yobs))=ℙ(max⁡Z(y)=max⁡Z(yobs))+ℙ(max⁡Z(y)>max⁡Z(yobs))≈ℙ(yobs)+ℙ(max⁡Z(y)≥max⁡Z(yobs);y≠yobs).
 MathType@MTEF@5@5@+=feaafiart1ev1aaatCvAUfKttLearuWrP9MDH5MBPbIqV92AaeXatLxBI9gBaebbnrfifHhDYfgasaacH8akY=wiFfYdH8Gipec8Eeeu0xXdbba9frFj0=OqFfea0dXdd9vqai=hGuQ8kuc9pgc9s8qqaq=dirpe0xb9q8qiLsFr0=vr0=vr0dc8meaabaqaciaacaGaaeqabaqabeGadaaakqaabeqaamrr1ngBPrwtHrhAYaqeguuDJXwAKbstHrhAGq1DVbaceaGae8xgHaLaeiikaGIagiyBa0MaeiyyaeMaeiiEaGNaeeOwaOLaeiikaGIaemyEaKNaeiykaKIaeyyzImRagiyBa0MaeiyyaeMaeiiEaGNaeeOwaOLaeiikaGIaemyEaK3aaSbaaSqaaiabd+gaVjabdkgaIjabdohaZbqabaGccqGGPaqkcqGGPaqkaeaacqGH9aqpcqWFzecucqGGOaakcyGGTbqBcqGGHbqycqGG4baEcqqGAbGwcqGGOaakcqWG5bqEcqGGPaqkcqGH9aqpcyGGTbqBcqGGHbqycqGG4baEcqqGAbGwcqGGOaakcqWG5bqEdaWgaaWcbaGaem4Ba8MaemOyaiMaem4CamhabeaakiabcMcaPiabcMcaPiabgUcaRiab=LriqjabcIcaOiGbc2gaTjabcggaHjabcIha4jabbQfaAjabcIcaOiabdMha5jabcMcaPiabg6da+iGbc2gaTjabcggaHjabcIha4jabbQfaAjabcIcaOiabdMha5naaBaaaleaacqWGVbWBcqWGIbGycqWGZbWCaeqaaOGaeiykaKIaeiykaKcabaGaeyisISRae8xgHaLaeiikaGIaemyEaK3aaSbaaSqaaiabd+gaVjabdkgaIjabdohaZbqabaGccqGGPaqkcqGHRaWkcqWFzecucqGGOaakcyGGTbqBcqGGHbqycqGG4baEcqqGAbGwcqGGOaakcqWG5bqEcqGGPaqkcqGHLjYScyGGTbqBcqGGHbqycqGG4baEcqqGAbGwcqGGOaakcqWG5bqEdaWgaaWcbaGaem4Ba8MaemOyaiMaem4CamhabeaakiabcMcaPiabcUda7iabdMha5jabgcMi5kabdMha5naaBaaaleaacqWGVbWBcqWGIbGycqWGZbWCaeqaaOGaeiykaKIaeiOla4caaaa@B4D0@

In other words, the probability of an observed value is separated from the probability of other sampled observations. Therefore, even if none of the sampled maxZ values are greater than the observed maxZ value, the corresponding *p*-value is nonzero.

**Importance sampling distribution** In order to avoid the problems of the MC procedure, it is natural to define the IS distribution such that it also gives observations from the upper tail of the maxZ distribution. We chose as an importance sampling distribution a mixture of the multinomial distribution

g*=ℙ(n1,n2,...,nJ|β10,β20,...,βJ0,α,ε)=α(nn1,0...nJ,0)∏i=1Jβj,00nj,0+1−αK∑k=1K(nn1,k...nJ,k)∏j=1Jβj,knj,k,     (10)
MathType@MTEF@5@5@+=feaafiart1ev1aaatCvAUfKttLearuWrP9MDH5MBPbIqV92AaeXatLxBI9gBaebbnrfifHhDYfgasaacH8akY=wiFfYdH8Gipec8Eeeu0xXdbba9frFj0=OqFfea0dXdd9vqai=hGuQ8kuc9pgc9s8qqaq=dirpe0xb9q8qiLsFr0=vr0=vr0dc8meaabaqaciaacaGaaeqabaqabeGadaaakeaafaqadeWabaaabaGaem4zaCMaeiOkaOIaeyypa0Zefv3ySLgznfgDOjdaryqr1ngBPrginfgDObcv39gaiqaacqWFzecucqGGOaakcqWGUbGBdaWgaaWcbaGaeGymaedabeaakiabcYcaSiabd6gaUnaaBaaaleaacqaIYaGmaeqaaOGaeiilaWIaeiOla4IaeiOla4IaeiOla4IaeiilaWIaemOBa42aaSbaaSqaaiabdQeakbqabaGccqGG8baFiiGacqGFYoGydaqhaaWcbaGaeGymaedabaGaeGimaadaaOGaeiilaWIae4NSdi2aa0baaSqaaiabikdaYaqaaiabicdaWaaakiabcYcaSiabc6caUiabc6caUiabc6caUiabcYcaSiab+j7aInaaDaaaleaacqWGkbGsaeaacqaIWaamaaGccqGGSaalcqGFXoqycqGGSaalcqGF1oqzcqGGPaqkaeaacqGH9aqpcqGFXoqycqGGOaakdaWcaaqaaiabd6gaUbqaaiabd6gaUnaaBaaaleaacqaIXaqmcqGGSaalcqaIWaamaeqaaOGaeiOla4IaeiOla4IaeiOla4IaemOBa42aaSbaaSqaaiabdQeakjabcYcaSiabicdaWaqabaaaaOGaeiykaKYaaebmaeaacqGFYoGydaqhaaWcbaGaemOAaOMaeiilaWIaeGimaadabaGaeGimaaJaemOBa42aaSbaaWqaaiabdQgaQjabcYcaSiabicdaWaqabaaaaaWcbaGaemyAaKMaeyypa0JaeGymaedabaGaemOsaOeaniabg+GivdaakeaacqGHRaWkdaWcaaqaaiabigdaXiabgkHiTiab+f7aHbqaaiabdUealbaadaaeWaqaaiabcIcaOmaalaaabaGaemOBa4gabaGaemOBa42aaSbaaSqaaiabigdaXiabcYcaSiabdUgaRbqabaGccqGGUaGlcqGGUaGlcqGGUaGlcqWGUbGBdaWgaaWcbaGaemOsaOKaeiilaWIaem4AaSgabeaaaaGccqGGPaqkdaqeWaqaaiab+j7aInaaDaaaleaacqWGQbGAcqGGSaalcqWGRbWAaeaacqWGUbGBdaWgaaadbaGaemOAaOMaeiilaWIaem4AaSgabeaaaaaaleaacqWGQbGAcqGH9aqpcqaIXaqmaeaacqWGkbGsa0Gaey4dIunaaSqaaiabdUgaRjabg2da9iabigdaXaqaaiabdUealbqdcqGHris5aOGaeiilaWcaaiaaxMaacaWLjaWaaeWaaeaacqaIXaqmcqaIWaamaiaawIcacaGLPaaaaaa@B4F7@

where 0 <*α *< 1,

βi,j={ε+(1−ε)βi0K,i=j,(1−ε)βi0K,i≠j,
 MathType@MTEF@5@5@+=feaafiart1ev1aaatCvAUfKttLearuWrP9MDH5MBPbIqV92AaeXatLxBI9gBaebbnrfifHhDYfgasaacH8akY=wiFfYdH8Gipec8Eeeu0xXdbba9frFj0=OqFfea0dXdd9vqai=hGuQ8kuc9pgc9s8qqaq=dirpe0xb9q8qiLsFr0=vr0=vr0dc8meaabaqaciaacaGaaeqabaqabeGadaaakeaaiiGacqWFYoGydaWgaaWcbaGaemyAaKMaeiilaWIaemOAaOgabeaakiabg2da9maaceqabaqbaeaabiGaaaqaaiab=v7aLjabgUcaRiabcIcaOiabigdaXiabgkHiTiab=v7aLjabcMcaPmaalaaabaGae8NSdi2aa0baaSqaaiabdMgaPbqaaiabicdaWaaaaOqaaiabdUealbaacqGGSaalaeaacqWGPbqAcqGH9aqpcqWGQbGAcqGGSaalaeaacqGGOaakcqaIXaqmcqGHsislcqWF1oqzcqGGPaqkdaWcaaqaaiab=j7aInaaDaaaleaacqWGPbqAaeaacqaIWaamaaaakeaacqWGlbWsaaGaeiilaWcabaGaemyAaKMaeyiyIKRaemOAaOMaeiilaWcaaaGaay5Eaaaaaa@578B@

and *n*_*j*,*k *_denotes the *j*th symbol frequency in the *k*th mixture. Notice that the number of the mixture components is the same as the number of the symbols plus one, i.e. *K *+ 1 = *J *+ 1.

The IS distribution consists of *K *+ 1 mixture components and two parameters *ε *and *α*. The first component ensures that some samples are drawn from the background distribution. The other *K *components correspond to the symbols, such that the probability of one symbol is high (≈ ε) and the probabilities of the other symbols are proportional to their background probabilities. The mixture parameter *α *determines which part of the samples are drawn from the background distribution and which from the other distributions. The shape parameter *ε *determines the weight for one of the symbols in each of the *K *mixture distributions.

This particular distribution was chosen for two reasons: first, if we choose a large enough *ε*, we can draw extreme observations from the 20 dimensional parameter space, and thus obtain large maxZ values; second, the use of mixture parameter *α *gives us a good coverage of the parameter space thereby ensuring that the process converges in a reasonable time. This is very important because the tests are made separately for each alignment position, and therefore the number of simulations made should be as low as possible.

**Importance sampling procedure** The following procedure summarizes the calculation of the observed significant levels using the importance sampling method.

1. Calculate observed maxZ value maxZ(*y*_*obs*_) using formula (7).

2. Choose parameter values *α *and *ε *for the IS distribution g* in formula (10) and the number of the samples *S*. Initialize ratio *r *= 0.

3. Generate a sample from distribution g* in formula (10) with the chosen *α *and *ε*.

4. Calculate maxZ(*y*) value for the sampled observation using formula (7).

5. If maxZ(*y*) ≥ maxZ(*y*_*obs*_), calculate ratio *r *= *r *+ *g*^*o*^(*y*_*i*_)/g* (*y*_*i*_). Otherwise do nothing.

6. Repeat the stages 3–5 *S *times.

7. Calculate the *p*-value according to the ratio *r*/*S*.

8. Output the -log(*p*) values.

**Parameter values** In the evaluation of the maxZ score, parameter values *α *= 0.4 and *ε *= 0.7 were used for the alignments with *N *≤ 100, and *α *= 0.4, *ε *= 0.8 for the remaining alignments. In the evaluation of the AQ score, *α *= 0.4 and *ε *= 0.7 were used for all benchmarking datasets. The number of the sample was *S *= 40,000 when evaluating the maxZ score, and *S *= 10,000 when calculating the quality of the alignments. After ensuring the sufficient accuracy of the procedure, the number of the samples was decreased in the alignment quality calculations, as there the objective was to define the number of the significant positions rather than the exact *p*-values. This enabled us to speed up the quality comparisons. The choice of the parameter values has been described in detail in the supplementary material (see Additional file 10). For the background distribution *g*^0^, we used the distribution of amino acids in the full alignment under investigation.

### The alignment quality (AQ) score

This section describes how the positional significance levels can be used to derive the alignment quality (AQ) score for the entire multiple sequence alignment. In the previous section, the conserved positions of the multiple sequence alignment were detected by the statistical hypothesis testing procedure. The significance tests were performed simultaneously at each alignment position. This simultaneous testing for the family of hypotheses causes a large multiple comparison problem which must be considered when deriving the quality score for the entire alignment.

Traditionally, these kinds of multiple comparison problems are solved by controlling the Family-Wise-Error-rate (FWE), i.e., the probability that at least one hypothesis is erroneously rejected [51]. Control of the FWE, however, decreases the probability of detecting the truly unconserved positions. Moreover, as several positions in the multiple sequence alignment can be considered to be conserved, a more natural approach is to control the False Discovery Rate (FDR), i.e., the expected proportion of the erroneously rejected null hypotheses. Controlling the expected proportion of rejected null hypotheses from the total number of the rejected hypotheses was first introduced by Benjamini and Hochberg [8].

Controlling the FDR led us to derive the alignment quality score following the Benjamini+Yekutieli's procedure [52]. More precisely, we use the following step-up procedure for controlling the FDR among arbitrarily dependent test statistics:

1. Calculate *p*-values for each alignment position. Let *P*_(1) _≤ *P*_(2) _≤ ... *P*_(*m*) _be the ordered list of the *p*-values.

2. Calculate j* = max{*j *: *P*_(*j*) _≤ jqm
 MathType@MTEF@5@5@+=feaafiart1ev1aaatCvAUfKttLearuWrP9MDH5MBPbIqV92AaeXatLxBI9gBaebbnrfifHhDYfgasaacH8akY=wiFfYdH8Gipec8Eeeu0xXdbba9frFj0=OqFfea0dXdd9vqai=hGuQ8kuc9pgc9s8qqaq=dirpe0xb9q8qiLsFr0=vr0=vr0dc8meaabaqaciaacaGaaeqabaqabeGadaaakeaadaWcaaqaaiabdQgaQjabdghaXbqaaiabd2gaTbaaaaa@30E7@}, where 0 <*q *< 1 is any fixed FDR and *m *is the length of the multiple sequence alignment.

3. If positive j* exists, choose positions associated with P_(1)_, *P*_(2)_, ..., *P*_(j*) _as conserved.

With the help of the number of the conserved positions j*, we obtain the proportion of conserved residues *ConsAA *by dividing the number of residues in the conserved positions by the number of residues in the whole alignment

ConsAA=∑i=1j∗ni∑i=1mni.     (11)
 MathType@MTEF@5@5@+=feaafiart1ev1aaatCvAUfKttLearuWrP9MDH5MBPbIqV92AaeXatLxBI9gBaebbnrfifHhDYfgasaacH8akY=wiFfYdH8Gipec8Eeeu0xXdbba9frFj0=OqFfea0dXdd9vqai=hGuQ8kuc9pgc9s8qqaq=dirpe0xb9q8qiLsFr0=vr0=vr0dc8meaabaqaciaacaGaaeqabaqabeGadaaakeaacqWGdbWqcqWGVbWBcqWGUbGBcqWGZbWCcqWGbbqqcqWGbbqqcqGH9aqpdaWcaaqaamaaqadabaGaemOBa42aaSbaaSqaaiabdMgaPbqabaaabaGaemyAaKMaeyypa0JaeGymaedabaGaemOAaOMaey4fIOcaniabggHiLdaakeaadaaeWaqaaiabd6gaUnaaBaaaleaacqWGPbqAaeqaaaqaaiabdMgaPjabg2da9iabigdaXaqaaiabd2gaTbqdcqGHris5aaaakiabc6caUiaaxMaacaWLjaWaaeWaaeaacqaIXaqmcqaIXaqmaiaawIcacaGLPaaaaaa@4EDE@

Here *n*_*i *_denotes the number of residues at the position *i*.

By comparing the *ConsAAs *calculated from the test and reference alignments, we can define the alignment quality *AQ *score as

*AQ *= [1 - (|*ConsAA*_*ref *_- *ConsAA*_*test*_|/*ConsAA*_*ref*_)] * 100.     (12)

The *AQ *score addresses to the question how many percent the test ConsAA is from the reference ConsAA. It is presumed that the higher the AQ value the better is the quality of the alignment. Note that the AQ score does not require the conserved positions to be the same in the test and reference alignments, only the number of the conserved residues counts.

#### Multiple sequence alignment programs

We compared the alignment quality of six multiple sequence alignment programs which have been widely used in bioinformatics: Clustal [9], TCoffee [10], Dialign2 [11], ProbCons[12], Muscle [13], and Mafft [14,15]. We used the default settings of the programs (Table 3). Out of 7 possible alignment strategies with Mafft, we chose L-INS-i, the most accurate method at the moment, and FFT-NS-2, the default method.

**Table 3:** Alignment programs and parameters used.

Program	Version	Parameters (strategy)
Clustal[9]	1.83	default
TCoffee[10]	2.66	default
Dialign2[11]	2.2.1	default
Probcons [12]	1.10	default
Muscle[13]	3.52	default
Mafft[14, 15]	5.667	-localpair -maxiterate 1000 (L-INS-i)
Mafft[14, 15]	5.667	default (FFT-NS-2)

#### Benchmarking database

We used the BAliBASE 3.0 database to test the alignment quality of the alignment programs. The BAliBASE is built for comparing alignment programs [45]. The database contains 218 multiple protein sequence alignments which have been divided into 5 reference sets. The reference set 1 includes equi-distant sequences, whose identity is less than 20% (ref 11) or between 20 and 40% (ref 12). The 2nd reference set consists of families aligned with a highly divergent "orphan" sequence. The 3rd reference set includes subgroups of sequences whose residue identity between the subgroups is less than 25%. The sequences of the 4th reference set contains N/C-terminal extensions. The 5th reference set consists of sequences with internal insertions.

Each alignment in the BAliBASE has two versions: one with full-length sequences and another with truncated sequences containing the sequences corresponding to the homologous regions only. We used only the truncated sequences, except in reference set 4, which only contains the full-length sequences. The BAliBASE annotates reliably aligned regions as core blocks. As in most of the studies using BAliBASE, we compared the alignment programs using the core block sequences only.

The BALiBASE provides a program called bali_score for calculating the SP and CS quality measures for the test alignment [1]. We used both of these scores for benchmarking.

#### Comparison procedure

In order to compare the quality of the alignments, the alignment programs were used to align each family in the BAliBASE database. The significance of the observed maxZ score was calculated for each alignment position. The proportion of conserved residues *ConsAA *was then calculated using 15 different FDRs varying from 0.01 to 0.15. The AQ score was calculated using the core blocks of each alignment. The core blocks of the new alignments were determined to consist of positions including one or more residues in the core block of the reference alignment. Additionally, the SP and CS scores were calculated for the core blocks using bali_score program.

The AQ, SP and CS scores were calculated for the 7 test alignments and the BAliBASE reference alignments for each set of sequences in the five reference sets. The results are presented as medians within each reference because the distributions of the AQ score values in the references were skewed. The statistical significance of the differences between the alignment programs were tested at FDR = 0.05 using Wilcoxon signed rank test. This test statistic was chosen because the same sequences were used in each method, and hence the scores could not be considered to be independent. The Bonferroni correction was used within each reference set to correct the effect of making multiple tests simultaneously. In the results section, the corrected p-values less than 0.05 were considered as statistically significant. For comparison of the relationship between the individual AQ and SP scores, we calculated the Spearman rank correlation coefficient separately for each 7 alignment methods.

## Authors' contributions

All authors participated in conceiving and designing the manuscript. VA, TA and EU carried out the theoretical considerations. VA performed the computational experiments and statistical analysis. VA, TA and MV drafted the manuscript. All authors read and approved the final manuscript.

## Supplementary Material

Additional File 1 **MultiDisp visualization and conservation scores for the Ras-like protein positions 1–31**. PNG formatted figure includes MultiDisp visualization of the Ras-like protein positions 1–31 (upper) and the corresponding conservation scores (lower). The curves show (red) the scaled -log(*p*)-values with Blosum62 scoring matrix, (magenta) the scaled -log(*p*)-values with grouping of amino acids, (blue) Mean Distance and (green) Information content scores for the alignment. Click here for file

Additional File 2 **MultiDisp visualization and conservation scores for the Ras-like protein positions 32–62**. As Additional file 1, but for the Ras-like protein positions 32–62. Click here for file

Additional File 3 **MultiDisp visualization and conservation scores for the Ras-like protein positions 63–92**. As Additional file 1, but for the Ras-like protein positions 63–92. Click here for file

Additional File 4 **MultiDisp visualization and conservation scores for the Ras-like protein positions 93–122**. As Additional file 1, but for the Ras-like protein positions 93–122. Click here for file

Additional File 5 **MultiDisp visualization and conservation scores for the Ras-like protein positions 123–152**. As Additional file 1, but for the Ras-like protein positions 123–152. Click here for file

Additional File 6 **MultiDisp visualization and conservation scores for the SH2 domain positions 1–27**. PNG formatted figure includes MultiDisp visualization of the SH2 domain positions 1–27 (upper) and the corresponding conservation scores (lower). The curves show (red) the scaled -log(*p*)-values with Blosum62 scoring matrix, (magenta) the scaled -log(*p*)-values with grouping of amino acids, (blue) Mean Distance and (green) Information content scores for the alignment. Click here for file

Additional File 7 **MultiDisp visualization and conservation scores for the SH2 domain positions 28–55**. As Additional file 6, but for SH2 domain positions 28–55. Click here for file

Additional File 8 **MultiDisp visualization and conservation scores for the SH2 domain positions 56–82**. As Additional file 6, but for SH2 domain positions 56–82. Click here for file

Additional File 9 **MultiDisp visualization and conservation scores for the SH2 domain positions 83–109**. As Additional file 6, but for SH2 domain positions 83–109. Click here for file

Additional File 10 **Tuning the importance sampling procedure**. PDF file describes the simulation procedure for finding the appropriate parameter values of the importance sampling procedure. Click here for file

## References

[B1] Thompson JD, Plewniak F, Poch O (1999). A comprehensive comparison of multiple sequence alignment programs. Nucleic Acids Res.

[B2] Karplus K, Hu BR (2001). Evaluation of protein multiple alignments by SAM-T99 using the BAliBASE multiple alignment test set. Bioinformatics.

[B3] Lassmann T, Sonnhammer ELL (2002). Quality assessment of multiple alignment programs. FEBS Lett.

[B4] O'Sullivan O, Zehnder M, Higgins D, Bucher P, Grosdidier A, Notredame C (2003). APDB: a novel measure for benchmarking sequence alignment methods without reference alignments. Bioinformatics.

[B5] Lassmann T, Sonnhammer ELL (2005). Automatic assessment of alignment quality. Nucleic Acids Res.

[B6] Gribskov M, McLachlan AD, Eisenberg D (1987). Profile analysis – detection of distantly related proteins. Proc Natl Acad Sci USA.

[B7] Rubin DB, Bernardo MH, an DeGroot KM, Lindley CV, Smith AFM (1988). Using the SIR algorithm to simulate posterior distributions. Bayesian Statistics 3.

[B8] Benjamini Y, Hochberg Y (1995). Controlling the false discovery rate – a practical and powerful approach to multiple testing. J R Stat Soc Ser B.

[B9] Thompson JD, Gibson TJ, Plewniak F, Jeanmougin F, Higgins DG (1997). The CLUSTAL_X windows interface: flexible strategies for multiple sequence alignment aided by quality analysis tools. Nucleic Acids Res.

[B10] Notredame C, Higgins DG, Heringa J (2000). T-Coffee: a novel method for fast and accurate multiple sequence alignment. J Mol Biol.

[B11] Morgenstern B (1999). DIALIGN 2: improvement of the segment-to-segment approach to multiple sequence alignment. Bioinformatics.

[B12] Do CB, Mahabhashyam MSP, Brudno M, Batzoglou S (2005). ProbCons: probabilistic consistency-based multiple sequence alignment. Genome Res.

[B13] Edgar RC (2004). MUSCLE: multiple sequence alignment with high accuracy and high throughput. Nucleic Acids Res.

[B14] Katoh K, Misawa K, Kuma K, Miyata T (2002). MAFFT: a novel method for rapid multiple sequence alignment based on fast Fourier transform. Nucleic Acids Res.

[B15] Katoh K, Kuma K, Toh H, Miyata T (2005). MAFFT version 5: improvement in accuracy of multiple sequence alignment. Nucleic Acids Res.

[B16] Gotoh O (1999). Multiple sequence alignment: algorithms and 
applications. Advances in Biophysics.

[B17] Valdar WSJ (2002). Scoring residue conservation. Proteins.

[B18] Sander C, Schneider R (1991). Database of homology-derived protein structures and the structural meaning of sequence alignment. Proteins.

[B19] Shenkin PS, Erman B, Mastrandrea LD (1991). Information-theoretical entropy as a measure of sequence variability. Proteins.

[B20] Hertz GZ, Stormo GD (1999). Identifying DNA and protein patterns with statistically significant alignments of multiple sequences. Bioinformatics.

[B21] Lawrence CE, Altschul SF, Boguski MS, Liu JS, Neuwald AF, Wootton JC (1993). Detecting subtle sequence signals – a Gibbs sampling strategy for multiple alignment. Science.

[B22] Taylor WR (1986). The classification of amino-acid conservation. J Theor Biol.

[B23] Zvelebil MJ, Barton GJ, Taylor WR, Sternberg MJE (1987). Prediction of protein secondary structure and active sites using the alignment of homologous sequences. J Mol Biol.

[B24] Mirny LA, Shakhnovich EI (1999). Universally conserved positions in protein folds: reading evolutionary signals about stability, folding kinetics and function. J Mol Biol.

[B25] Livingstone CD, Barton GJ (1993). Protein-sequence alignments – a strategy for the hierarchical analysis of residue conservation. Comput Appl Biosci.

[B26] Henikoff S, Henikoff JG (1993). Performance evaluation of amino-acid substitution matrices. Proteins.

[B27] Benner SA, Cohen MA, Gonnet GH (1994). Amino-acid substitution during functionally constrained divergent evolution of protein sequences. Protein Eng.

[B28] Dayhoff MO, Schwartz RM, Orcutt BC, Dayhoff MO (1978). A model of evolutionary change in proteins. Atlas of protein sequence and structure.

[B29] Carrillo H, Lipman D (1988). The multiple sequence alignment problem in biology. SIAM J Appl Math.

[B30] Thompson JD, Plewniak F, Ripp R, Thierry JC, Poch O (2001). Towards a reliable objective function for multiple sequence alignments. J Mol Biol.

[B31] Pei JM, Grishin NV (2001). AL2CO: calculation of positional conservation in a protein sequence alignment. Bioinformatics.

[B32] Ahola V, Aittokallio T, Uusipaikka E, Vihinen M (2004). Statistical methods for identifying conserved residues in multiple sequence alignment. Stat Appl Genet Mol Biol.

[B33] Ahola V, Aittokallio T, Uusipaikka E, Vihinen M (2003). Efficient estimation of emission probabilities in profile hidden Markov models. Bioinformatics.

[B34] Bateman A, Coin L, Durbin R, Finn RD, Hollich V, Griffiths-Jones S, Khanna A, Marshaff M, Moxon S, Sonnhammer ELL, Studholme DJ, Yeats C, Eddy SR (2004). The Pfam protein families database. Nucleic Acids Res.

[B35] Oliveira L, Paiva PB, Paiva ACM, Vriend G (2003). Identification of functionally conserved residues with the use of entropy-variability plots. Proteins.

[B36] Oliveira L, Paiva ACM, Vriend G (1993). A common motif in G-protein-coupled 7 transmembrane helix receptors. J Comput Aided Mol Des.

[B37] MultiDisp graphics program. http://bioinf.uta.fi/cgi-bin/MultiDisp.cgi.

[B38] Shen B, Vihinen M (2004). Conservation and covariance in PH domain sequences: physicochemical profile and information theoretical analysis of XLA-causing mutations in the Btk PH domain. Protein Eng Des Sel.

[B39] Songyang Z, Shoefson SE, Chaudhuri M, Gish G, Pawson T, Haser WG, King F, Roberts T, Ratnofsky S, Lechleider RJ, Neel BG, Birge RB, Fajardo JE, Chou MM, Hanafusa H, Schaffhausen B, Cantley LC (1993). SH2 domains recognize specific phosphopeptide sequences. Cell.

[B40] Pawson T, Gish GD, Nash P (2001). SH2 domains, interaction modules and cellular wiring. Trends Cell Biol.

[B41] Bradshaw JM, Waksman G (2002). Molecular recognition by SH2 domains. Adv Protein Chem.

[B42] Waksman G, Shoelson SE, Pant N, Cowburn D, Kuriyan J (1993). Binding of a high-affinity phosphotyrosyl peptide to the Src Sh2 domain – crystal-structures of the complexed and peptide-free forms. Cell.

[B43] Overduin M, Rios CB, Mayer BJ, Baltimore D, Cowburn D (1992). 3-Dimensional solution structure of the Src homology-2 domain of C-Abl. Cell.

[B44] Bianchetti L, Oudet C, Poch O (2002). M13 endopeptidases: new conserved motifs correlated with structure, and simultaneous phylogenetic occurrence of PHEX and the bony fish. Proteins.

[B45] Bahr A, Thompson JD, Thierry JC, Poch O (2001). BAliBASE (Benchmark Alignment dataBASE): enhancements for repeats, transmembrane sequences and circular permutations. Nucleic Acids Res.

[B46] Ott J (1979). Maximum likelihood estimation by counting methods under polygenic and mixed models in human pedigrees. Am J Hum Genet.

[B47] Kong A, Frigge M, Irwin M, Cox N (1992). Importance sampling *I*: Computing multimodel-P values in linkage analysis. Am J Hum Genet.

[B48] mafft 5.7. http://www.biophys.kyoto-u.ac.jp/~katoh/programs/align/mafft/.

[B49] Bernard GA (1963). Discussion of paper by MS Bartlett. J R Stat Soc Ser B.

[B50] Smith AFM, Gelfand AE (1992). Bayesian statistics without tears – a sampling resampling perspective. American Statistician.

[B51] Hochberg Y, Tamhane AC (1987). Multiple comparison procedures.

[B52] Benjamini Y, Yekutieli D (2001). The control of the false discovery rate in multiple testing under dependency. Annals of Statistics.

