# Supplementary figures and images for: A statistical score for assessing the quality of multiple sequence alignments
Source: BMC Bioinformatics. 2006 Nov 3;7:484. doi: 10.1186/1471-2105-7-484 (PMC1687212; doi:10.1186/1471-2105-7-484)

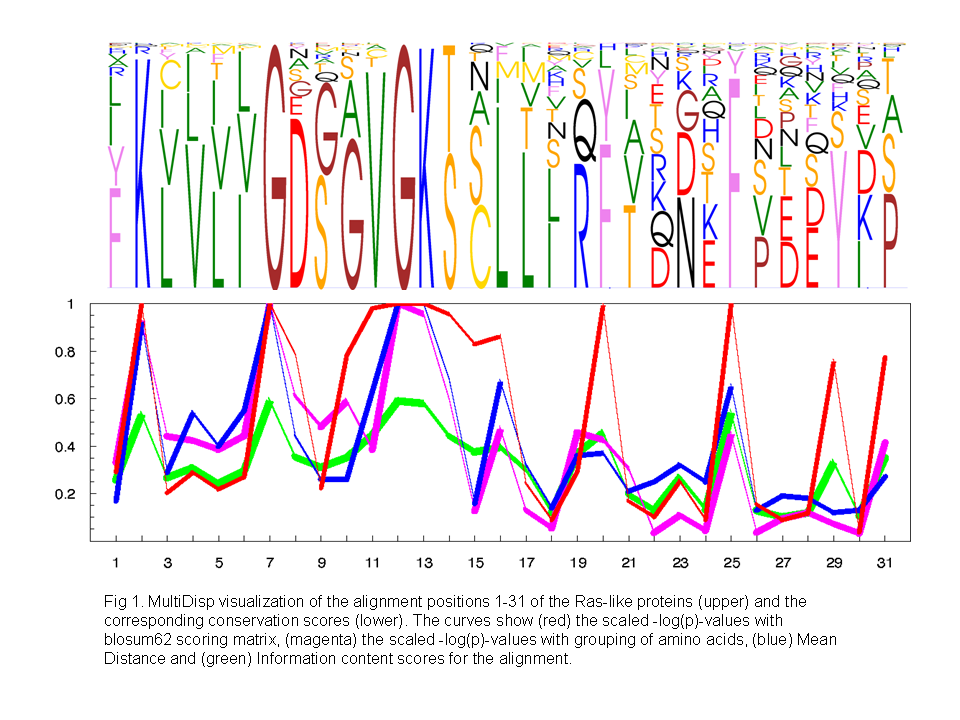

Supplement: Additional File 1 — MultiDisp visualization and conservation scores for the Ras-like protein positions 1–31. PNG formatted figure includes MultiDisp visualization of the Ras-like protein positions 1–31 (upper) and the corresponding conservation scores (lower). The curves show (red) the scaled -log(p)-values with Blosum62 scoring matrix, (magenta) the scaled -log(p)-values with grouping of amino acids, (blue) Mean Distance and (green) Information content scores for the alignment. [file 1471-2105-7-484-S1.png]

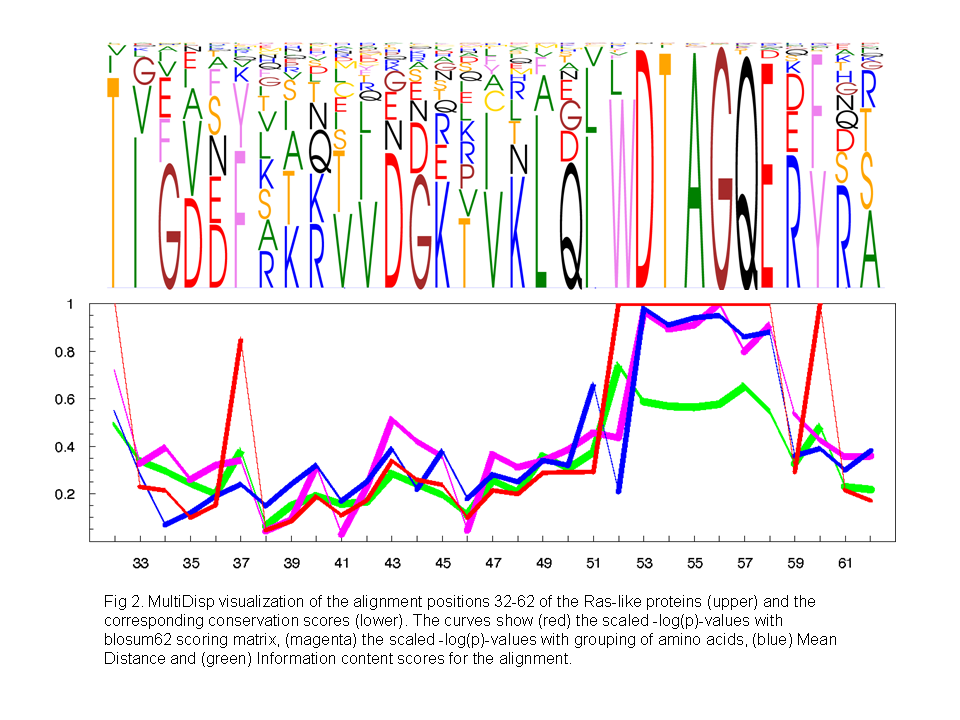

Supplement: Additional File 2 — MultiDisp visualization and conservation scores for the Ras-like protein positions 32–62. As Additional file 1, but for the Ras-like protein positions 32–62. [file 1471-2105-7-484-S2.png]

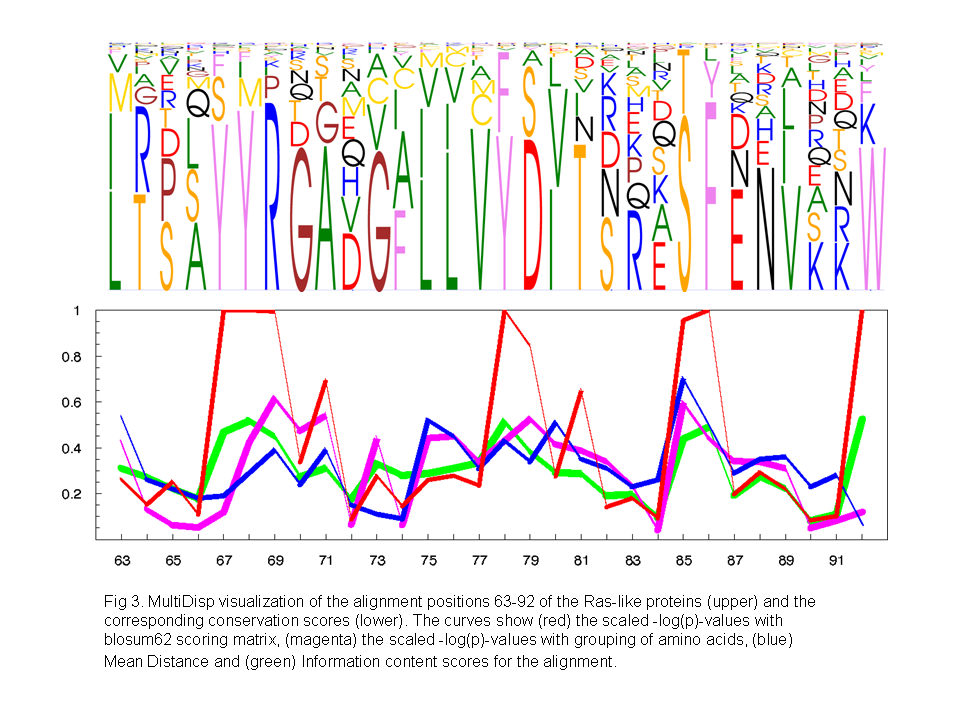

Supplement: Additional File 3 — MultiDisp visualization and conservation scores for the Ras-like protein positions 63–92. As Additional file 1, but for the Ras-like protein positions 63–92. [file 1471-2105-7-484-S3.png]

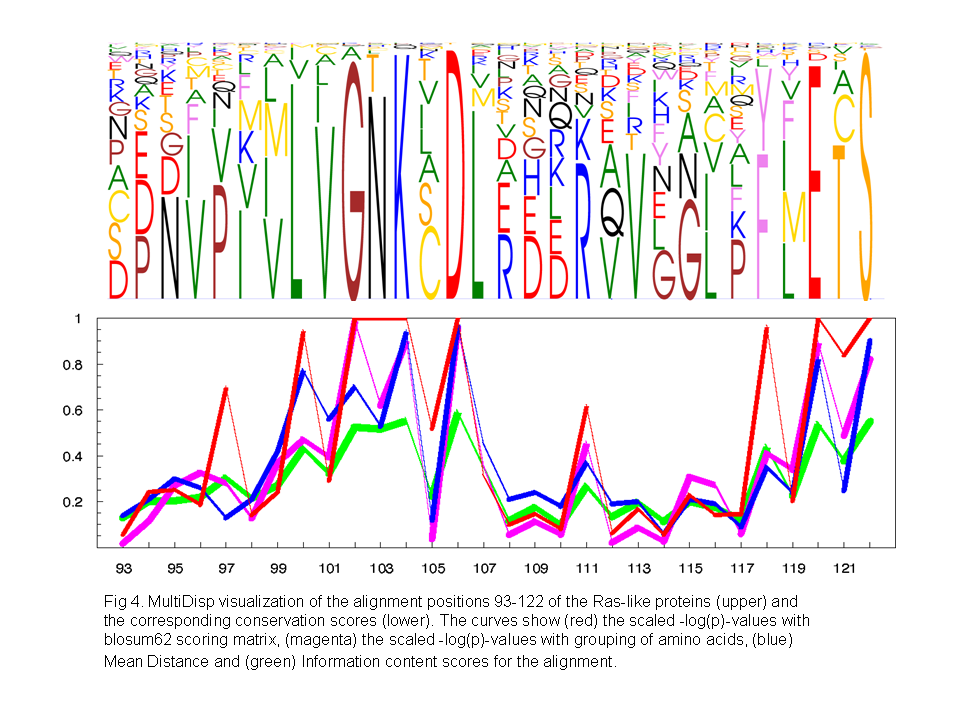

Supplement: Additional File 4 — MultiDisp visualization and conservation scores for the Ras-like protein positions 93–122. As Additional file 1, but for the Ras-like protein positions 93–122. [file 1471-2105-7-484-S4.png]

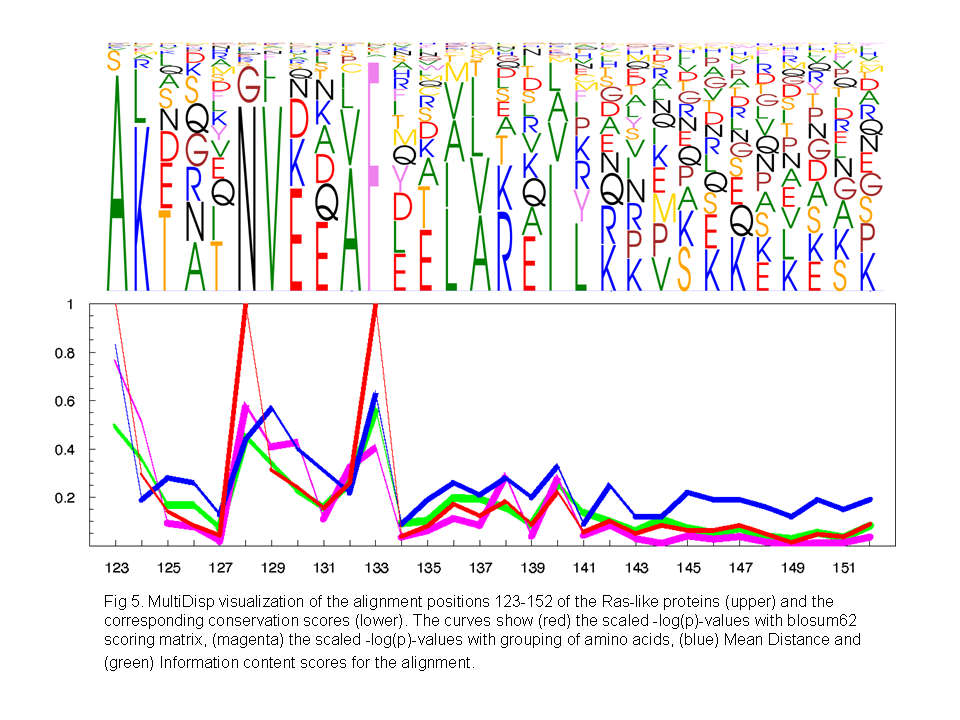

Supplement: Additional File 5 — MultiDisp visualization and conservation scores for the Ras-like protein positions 123–152. As Additional file 1, but for the Ras-like protein positions 123–152. [file 1471-2105-7-484-S5.png]

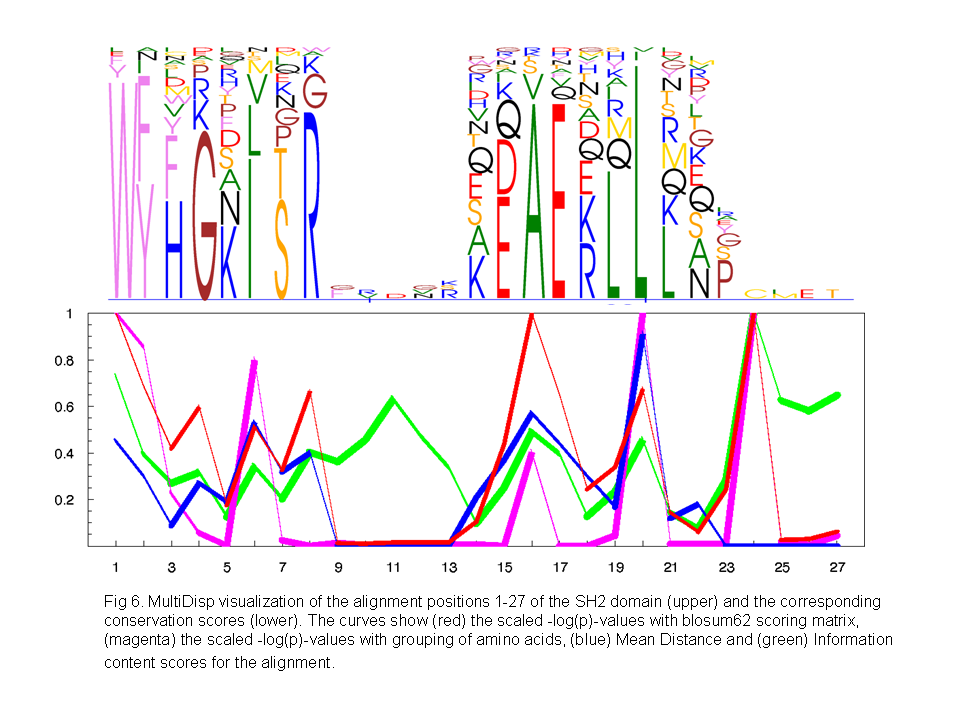

Supplement: Additional File 6 — MultiDisp visualization and conservation scores for the SH2 domain positions 1–27. PNG formatted figure includes MultiDisp visualization of the SH2 domain positions 1–27 (upper) and the corresponding conservation scores (lower). The curves show (red) the scaled -log(p)-values with Blosum62 scoring matrix, (magenta) the scaled -log(p)-values with grouping of amino acids, (blue) Mean Distance and (green) Information content scores for the alignment. [file 1471-2105-7-484-S6.png]

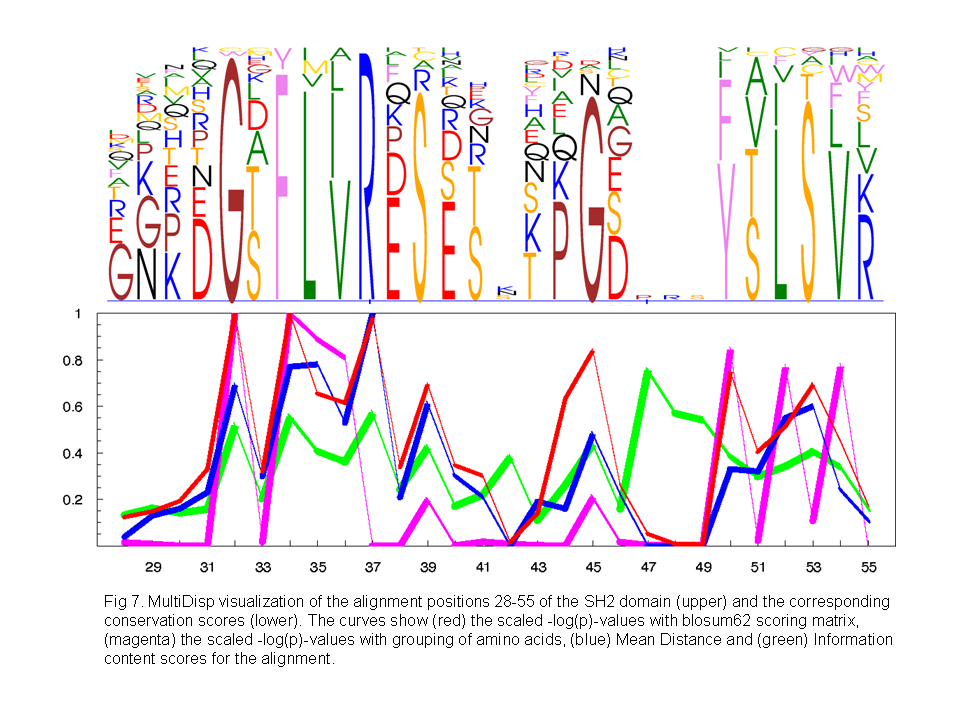

Supplement: Additional File 7 — MultiDisp visualization and conservation scores for the SH2 domain positions 28–55. As Additional file 6, but for SH2 domain positions 28–55. [file 1471-2105-7-484-S7.png]

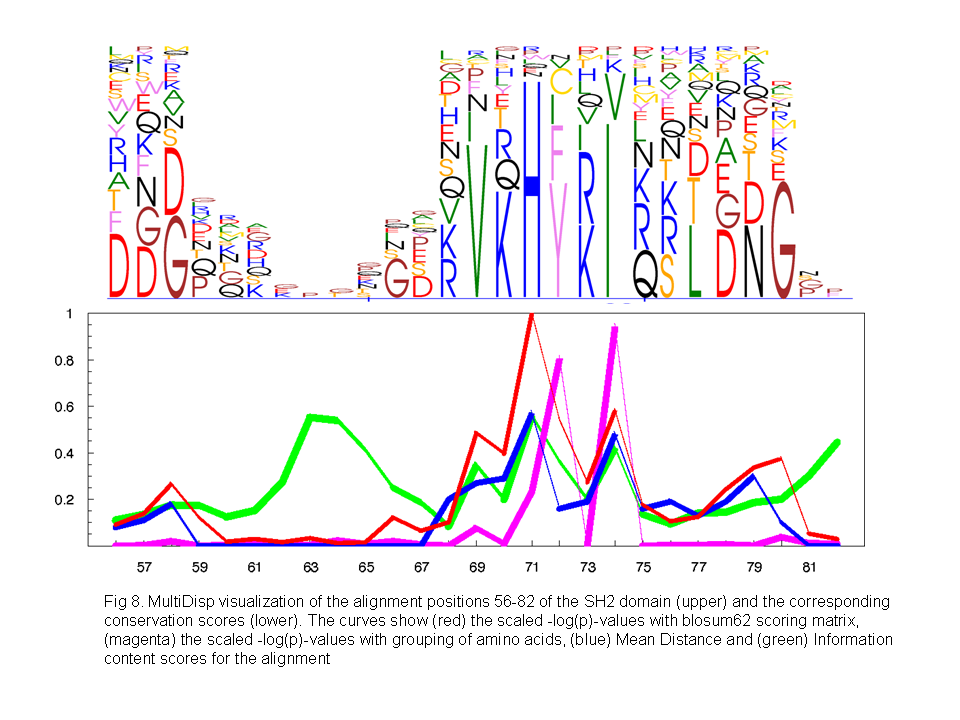

Supplement: Additional File 8 — MultiDisp visualization and conservation scores for the SH2 domain positions 56–82. As Additional file 6, but for SH2 domain positions 56–82. [file 1471-2105-7-484-S8.png]

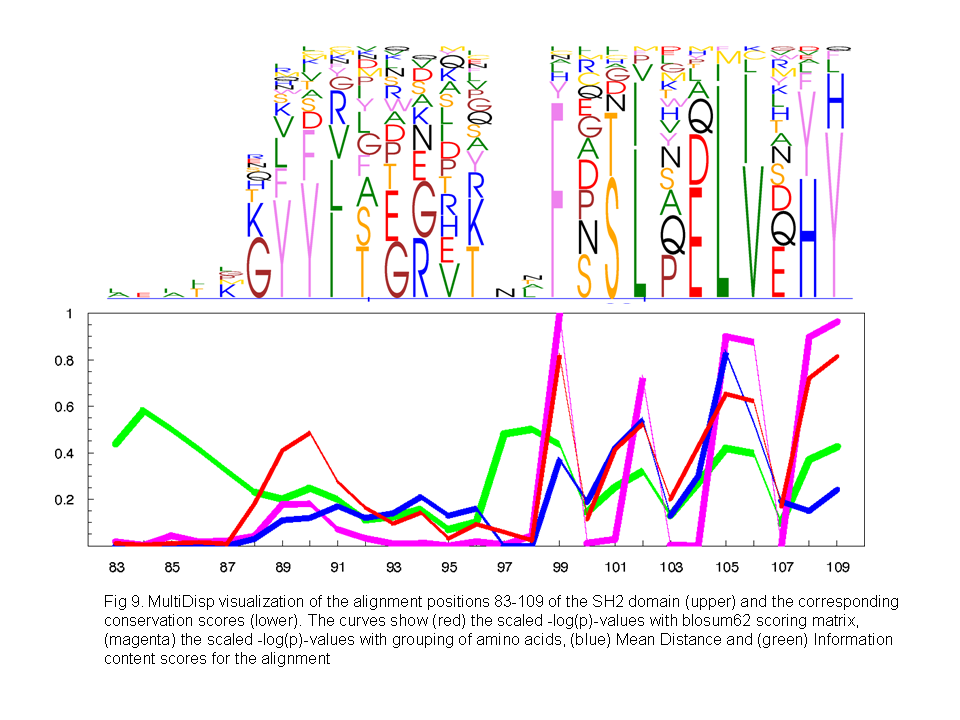

Supplement: Additional File 9 — MultiDisp visualization and conservation scores for the SH2 domain positions 83–109. As Additional file 6, but for SH2 domain positions 83–109. [file 1471-2105-7-484-S9.png]
