# Supplementary material for: A statistical score for assessing the quality of multiple sequence alignments
Source: BMC Bioinformatics. 2006 Nov 3;7:484. doi: 10.1186/1471-2105-7-484 (PMC1687212; doi:10.1186/1471-2105-7-484)
Supplement: Additional File 10 — Tuning the importance sampling procedure. PDF file describes the simulation procedure for finding the appropriate parameter values of the importance sampling procedure. [file 1471-2105-7-484-S10.pdf]

1 Tuning the importance sampling procedure

In this section, we study the choice of parameter values α and ϵ, and how the number of the sequences in the alignment affects this selection. Finally, we discuss how many samples are needed for the proper estimation of the p-values. The aim is to choose such parameter values that the variation between positions with different degrees of conservation can be distinguished, while the number of the simulations remains as low as possible.

Before seeking the appropriate parameter values for calculating the p-values, we tested the IS distribution by using the probability of an amino acid j as a test statistic i.e. by replacing $t(y)$ in equation (9) by $t(y) = I_{\{y=j\}}$. We then drew samples from the IS distribution with various α and ϵ values and calculated the IS probability (9), which should give the background probability of the amino acid j. We performed the test for alanine, lysine and arginine. When the number of the sequences was 200, the procedure converged to the correct value already in approximately 20,000 simulations; with 50 sequences the procedure converged in 40,000 simulations. The convergence was most rapid when both α and ϵ were close to 0.5.

Choosing the parameter values for the calculation of the observed significance level is more complicated than it was with the test statistic of the previous chapter, since the correct *p*-values were not known. We calculated *p*-values for three alignment positions: 1) very heterogenious, 2) moderately conserved and 3) highly conserved. The relative frequencies of the positions were, in fact, equivalent to 28th, 36th, and 34th alignment positions of the Pfam alignment of the SH2 domain [1]. The number of the sequences in the alignments were: 25, 50, 100, 200 or 300. Altogether 100,000 samples were taken from the importance sampling distribution where α was between 0.3 and 0.7 and ϵ varied from 0.5 to 0.9. Simulations were performed using the IS procedure.

Supplementary Figure 1 shows the negative logarithm of the p-values for three types of distributions (columns) and five different numbers of sequences (rows) as the function of the number of simulations. In the highly conserved position with $n = 200$, for all simulated observations y, $\max\mathrm{Z}(y) < \max\mathrm{Z}(y_{obs})$, and therefore the p-value is equal to the probability of an observation $P(y_{obs})$ at each simulation number. The parameter values were chosen such that all the three simulation examples converged as rapidly as possible. We suggest that when the number of the sequences in the alignment is 100 or less, $\alpha = 0.4$ and $\epsilon = 0.7$ are appropriate parameter values. For the alignments

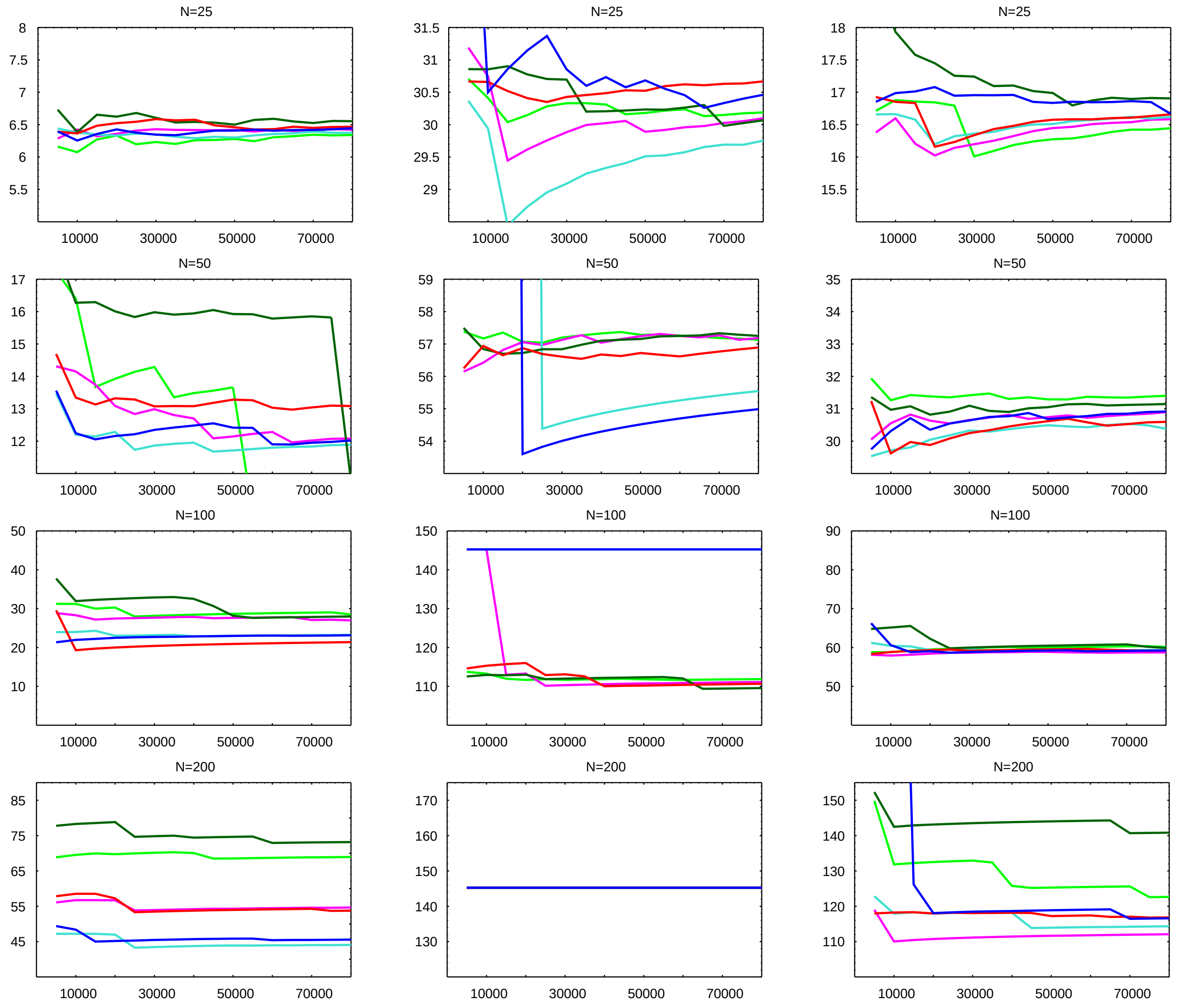

Figure 1: **-Log(*p*)-values as a function of the number of the simulations.** The columns represent heterogeneous, highly conserved and moderately conserved positions with different number of sequences: n=25, 50, 100 and 200 (rows). Curves indicate combinations of the parameter value of the IS distribution: $\alpha = 0.3, \epsilon = 0.6$, $\alpha = 0.3, \epsilon = 0.7$, $\alpha = 0.3, \epsilon = 0.8$, $\alpha = 0.6, \epsilon = 0.6$, $\alpha = 0.6, \epsilon = 0.7$ and $\alpha = 0.6, \epsilon = 0.8$.

with more than 100 sequences, $\epsilon = 0.8$ is better choice. With these α and ϵ values, the process will converge in ca. 40,000 simulation runs. When the number of sequences in the alignment is large (more than 100), 20,000 simulations are usually enough for the IS procedure to converge.

References

[1] Bateman,A., Coin,L., Durbin,R., Finn,R.D., Hollich,V., Griffiths-Jones,S., Khanna,A., Marshall,M., Moxon,S., Sonnhammer,E.L.L., Studholme,D.J., Yeats,C. and Eddy,S.R. (2004) *Nucleic Acids Res*, **32**, D138-D141.
